# Effect of multiple cyclic RGD peptides on tumor accumulation and intratumoral distribution of IRDye 700DX-conjugated polymers

**DOI:** 10.1038/s41598-018-26593-0

**Published:** 2018-05-25

**Authors:** Xuebo Dou, Takahiro Nomoto, Hiroyasu Takemoto, Makoto Matsui, Keishiro Tomoda, Nobuhiro Nishiyama

**Affiliations:** 1 Laboratory for Chemistry and Life Science, Institute of Innovative Research, Tokyo Institute of Technology, 4259 Nagatsuta-cho, Midori-ku, Yokohama, Kanagawa 226-8503 Japan; 2 Innovation Center of Nanomedicine (iCONM), Kawasaki Institute of Industrial Promotion, 3-25-14 Tonomachi, Kawasaki-ku, Kawasaki, Kanagawa 210-0821 Japan

## Abstract

Strategic delivery of IRDye 700DX (photosensitizer) is a key for improving its effect in photodynamic therapy. In this study, we have synthesized IRDye 700DX-conjugated polymers containing multiple cyclic RGD peptides to deliver IRDye 700DX selectively to tumor cells and tumor-associated blood vessels overexpressing α_v_β_3_ integrin. Our polymer has a backbone of hydrophilic poly(ethylene glycol)-poly(L-glutamic acid) block copolymer, and cyclic RGD peptides are conjugated to side chains of the poly(L-glutamic acid) while IRDye 700DX is conjugated to the terminal of poly(ethylene glycol). The polymers exhibited selective accumulation to the target sites in a subcutaneous solid tumor, and the accumulation was augmented with the increased number of cyclic RGD peptides. More importantly, the polymer containing 15 cyclic RGD peptides in one construct revealed preferential accumulation on the tumor-associated blood vessels without compromising penetration to deep portions of the tumor, thereby drastically inhibiting tumor growth upon photoirradiation, while the polymer containing 5 cyclic RGD peptides showed moderate antitumor activity despite efficient accumulation in the tumor with almost homogenous intratumoral distribution. These results suggest that controlling the intratumoral distribution of IRDye 700DX is critical for successful PDT, and our polymer containing multiple cyclic RGD peptides may be a promising carrier for this spatial control.

## Introduction

Photodynamic therapy (PDT) for cancer treatment is a therapeutic modality using light to activate a non-toxic photosensitizer (PS) to generate cytotoxic reactive oxygen species (ROS) that result in tissue devastation by direct killing of cancer cells, shutdown of microvasculature, and activation of immune systems^1^. Because of the selectivity, PDT has been receiving greater attention in recent years and has now become an important field in medical research^2^. Although numerous agents can be used as PSs, few PSs can be chosen as candidates for clinical application, because most PSs have some drawbacks including hydrophobicity, low photostability, and poor tumor-specificity, which may cause untoward damage to noncancerous cells and limit the application of PDT^3^.

An emerging PS, IRDye 700DX (700DX) has attracted increased attentions for its hydrophilicity, high photostability, and strong near-infrared radiation absorption^4^. Since it also lacks tumor-specificity, 700DX has been conjugated to antibodies to improve the accumulation into a tumor^5–10^. Antibody-700DX conjugates accumulated selectively within target tumors and showed phototoxicity only when it was bound to its target cells, illustrating that their minimal toxicity to normal tissues^6^. Because of these excellent properties, the antibody-700DX conjugates demonstrated their validity in the treatment of breast cancers^7^, lung metastases^8^, gastric cancers^9^ and programmed death-ligand 1-expressing tumors^10^. It is noteworthy that an antibody-700DX conjugate, RM-1929, is ongoing Phase II clinical trial for recurrent head and neck cancer (NCT02422979).

In spite of the outstanding specificity, large size and too strong affinity of antibodies sometimes limit their penetration into deep portions of tumors especially in poorly vascularized tumors, and inhomogeneous antigen expression in a tumor tissue leads to the heterogeneous distribution of the antibodies^11^, overshadowing successful PDT. As one of the approaches to overcome such tumor heterogeneity, Kobayashi and coworkers proposed super enhanced permeability and retention effect, in which antibody-700DX conjugates accumulating in a perivascular region in a tumor can destruct the corresponding tissue and increase the vascular permeability in the tumor^12^. Subsequent additional tumor accumulation and enhanced penetration of antibody-700DX conjugates could improve antitumor efficacy by repetitive photoirradiation^13^.

Meanwhile, to improve such PDT efficacy compromised by tumor heterogeneity, targeting tumor-associated vasculature may also offer a promising approach, because PDT extirpates tumors by not merely directly killing cancer cells but also shutting down the vasculature, which allows for the eradication of deep regions of a tumor even if PSs show inhomogeneous intratumoral distribution^3^. In this regard, a cyclic RGD (cRGD) peptide has great potential for targeting the tumor-associated vasculature^14^. The cRGD peptide specifically binds to α_v_β_3_ integrin, which is known to be overexpressed on activated endothelial cells of growing vessels and also on melanoma, glioma, lung, ovarian and breast cancer cells^15^. However, the applicability of the monomeric cRGD peptide has been limited by relatively low affinity compared with an antibody and eventual rapid washout from the tumor. One strategy for overcoming these limitations is the application of multiple cRGD peptides to improve the affinity to the α_v_β_3_ integrin^16–19^. It has been reported that multiple cRGD can increase the affinity to α_v_β_3_ integrin by multivalent effect^18^ and cluster effect^20^; previous studies indeed demonstrated that introduction of multiple cRGD peptides to a delivery system could enhance the affinity to α_v_β_3_ integrin and prevent the rapid washout from a target tumor^17,21^. In addition, cRGD peptides have been reported to activate integrin-mediated transcytosis that should enhance tumor penetration of delivery systems^22,23^. These unique properties of the cRGD peptide are expected to augment the therapeutic efficacy of 700DX by controlling its intratumoral distribution.

**Figure 1:**
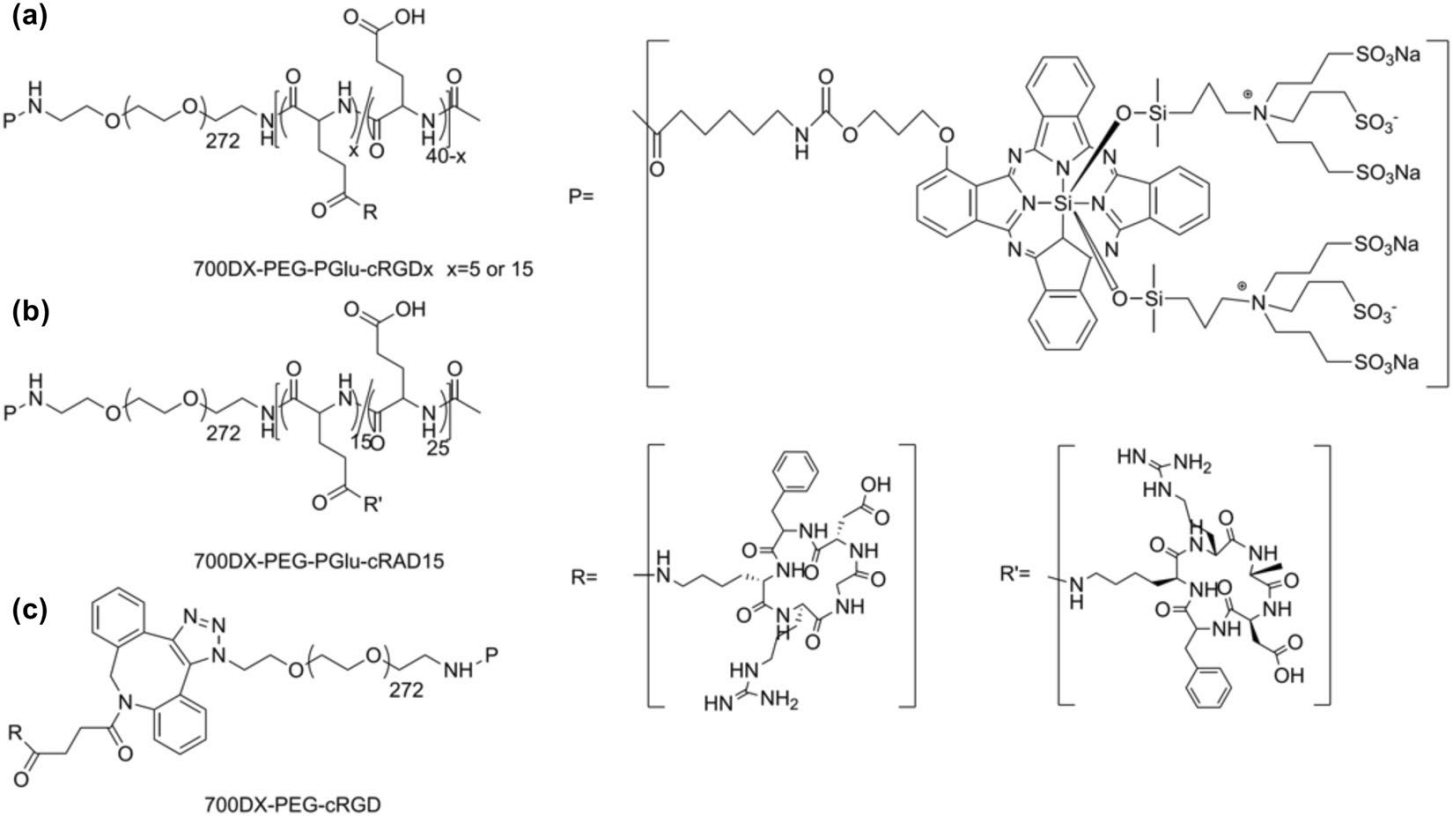
Schematic diagram illustrating the chemical structure of (**a**) 700DX-PEG-PGlu-cRGDx, (**b**) 700DX-PEG-PGlu-cRAD15, and (**c**) 700DX-PEG-cRGD.

Here, to deliver 700DX selectively to the tumor-associated vasculature as well as the target tumor cells with homogeneous intratumoral distribution and prolonged retention, we synthesized a 700DX-polymer conjugate having multimeric cRGD peptides (Fig. 1a). The 700DX-polymer conjugates exhibited higher cellular uptake and phototoxicity in α_v_β_3_ integrin-overexpressing cells than free 700DX, and these biological activities of the 700DX-polymer conjugates were augmented by the increased number of cRGD peptides. *In vivo* study revealed that multiple cRGD peptides moiety not only offered the selective delivery of 700DX to a subcutaneous tumor, but also prolonged the tumor retention, averting the rapid washout unlike monomeric cRGD peptide. More importantly, the multiple cRGD peptides permitted appreciable penetration in tumor tissue and preferential accumulation in the tumor-related blood vessels, and this preference became more notable by increasing the number of cRGD. This more preferential accumulation in tumor-associated vasculature drastically enhanced PDT effect of 700DX. Our results indicate that intratumoral distribution of 700DX critically affects the therapeutic outcome of 700DX, and this distribution could be controlled by fine-tuning the number of cRGD peptides in the 700DX-polymer conjugate. Our polymer conjugate offers a novel approach to attain strong *in vivo* therapeutic effect of 700DX.

## Results

### Synthesis and characterization of polymer conjugated photosensitizers

Figure 1a shows chemical structure of the 700DX-polymer conjugate having multimeric cRGD peptides, termed 700DX-PEG-PGlu-cRGDx (x denotes the number of cRGD peptides in the polymer). To synthesize this polymer conjugate, first, azide functionalized poly(ethylene glycol)-poly(γ-benzyl-L-glutamate) (azide-PEG-PBLG, molecular weight of PEG: 12,000, polymerization degree of PBLG: 40, Mw/Mn = 1.10) was prepared via ring-opining polymerization of γ-benzyl-L-glutamate *N*-carboxyanhydride (BLG-NCA) initiated by azide-functionalized PEG having a terminated amine group (azide-PEG-NH_2_), followed by protection of the ω-end amine group with acetic anhydride and deprotection of benzyl groups at the side chains to produce azide-PEG-poly(L-glutamic acid) (Supplementary Figure S1). Then, cRGD peptides were introduced to azide-PEG-poly(L-glutamic acid) (PEG-PGlu) via activation of the carboxyl groups in the side chain of PGlu with 1,1′-carbonyldiimidazole (CDI), and subsequent formation of amide groups with the amine groups of cyclic RGDfK (cRGDfK) peptides (Supplementary Figure S2). After reducing the azide group of PEG into the amine group, 700DX-*N*-hydroxysuccinimidyl ester (700DX-NHS) was conjugated to the polymer to obtain 700DX-PEG-PGlu-cRGDx (Supplementary Figure S3). As a control, a 700DX-polymer conjugate having 15 cyclic RADfK (cRADfK) peptides, which is termed 700DX-PEG-PGlu-cRAD15 (Fig. 1b), was synthesized in a similar manner, using cRADfK peptides instead of cRGDfK peptides (Supplementary Figures S4 and S5). Note that the cRAD peptide does not possess affinity to the α_v_β_3_ integrin^24^. To synthesize a monomeric cRGD peptide-functionalized PEG-700DX conjugate (700DX-PEG-cRGD) as another control (Fig. 1c), the cRGD peptide was first reacted with dibenzocyclooctyne-*N*-hydroxysuccinimidyl ester (DBCO-NHS). DBCO functionalized cRGD peptide was introduced to azide-PEG-NH_2_ via copper-free click chemistry (Supplementary Figure S6), and subsequently, 700DX-NHS was reacted with the amino group (Supplementary Figure S7). Quantitative conjugation of cRGD or cRAD and narrow molecular weight distribution were confirmed by ^1^H NMR and size exclusion chromatography, respectively (Supplementary Figures S8–S15). All the polymers showed narrow molecular weight distribution similarly with that of PEG-PGlu. 700DX-PEG-cRGD exhibited delayed retention time compared with PEG-PGlu due to lack of PGlu segment, while 700DX-PEG-PGlu-cRGD5, 700DX-PEG-PGlu-cRGD15, and 700DX-PEG-PGlu-cRAD15 revealed slightly earlier retention time, which is consistent with the increase of molecular weight by the conjugation of multiple cRGD peptides. It should be noted that 700DX-PEG-PGlu-cRGD5 and 700DX-PEG-PGlu-cRGD15 showed apparently different retention time, illustrating the fine-tuned number of the conjugated cRGD peptides. Also, we measured fluorescence spectra of the synthesized 700DX-conjugated polymers (Supplementary Figure S16). All the polymers emitted the similar fluorescence with 700DX, suggesting that the conjugation of 700DX to the polymers did not affect its photoactivity.

### Cellular uptake and subcellular distribution

**Figure 2:**
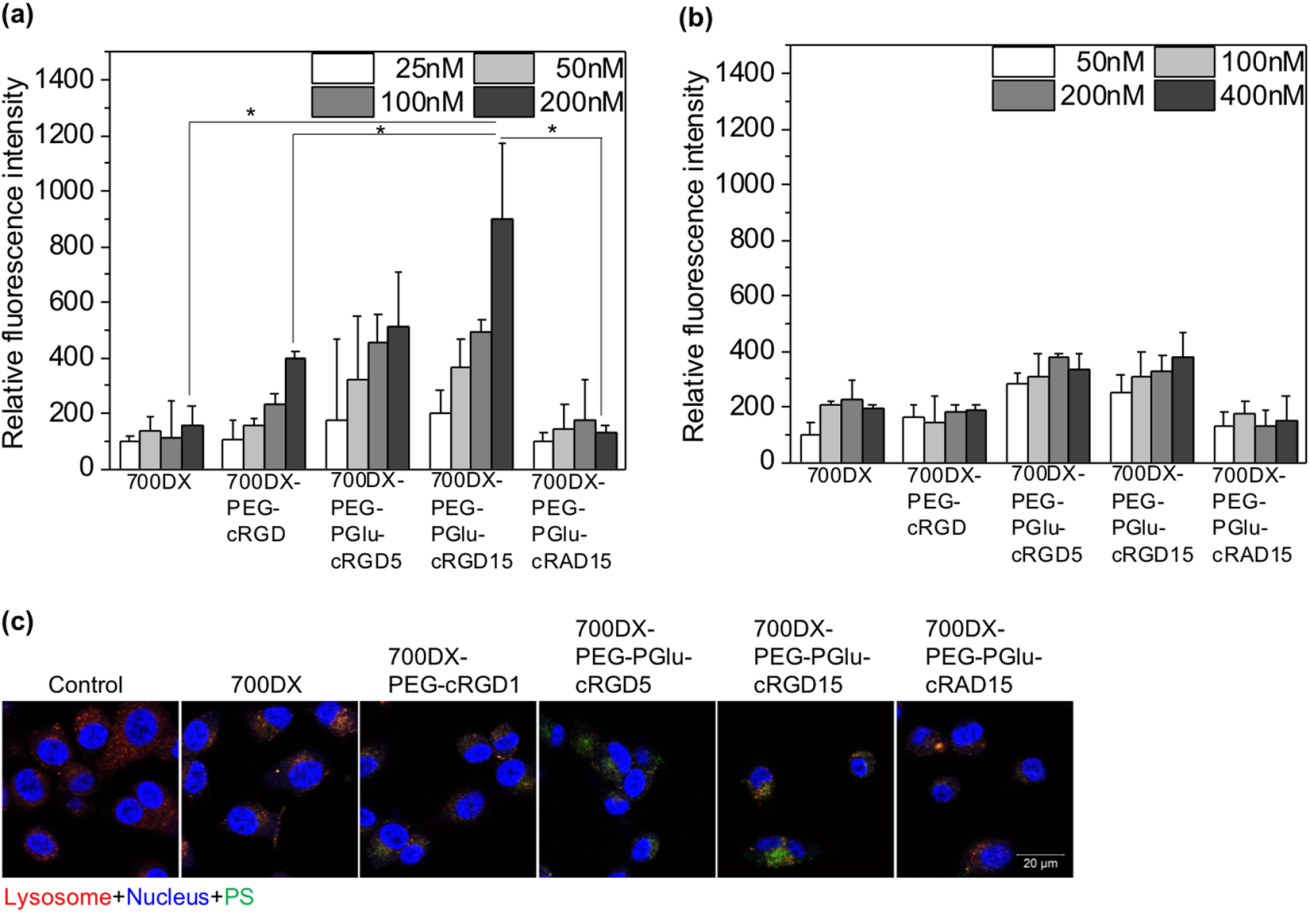
(**a**,**b**) Cellular uptake study. Cellular uptake was evaluated by measuring fluorescence intensity of PSs in (**a**) U87MG and (**b**) K562 cells. U87MG and K562 cells were incubated with the samples at various 700DX equivalent concentrations for 7 h before measuring the fluorescence intensity (Ex = 710 nm, Em = 780 nm). The results are expressed as mean ± S.D. (n = 6). Statistical analysis was performed using one-way ANOVA at 200 nM (*p < 0.01). (**c**) Subcellular distribution of PSs in U87MG cells. After 7 h incubation with PSs, fixed cells were observed using CLSM. The fluorescence of 700DX, Lysotracker Red DND-99, and Hoechst33342 are shown in green, red, and blue, respectively.

The *in vitro* cellular uptake of the PSs was assessed by measuring the fluorescence intensity of PSs in U87MG (human glioblastoma) cells overexpressing α_v_β_3_ integrin and K562 (human myelogenous leukemia) cells with low expression level of α_v_β_3_ integrin^25^, using flow cytometry. Figure 2a shows cellular uptake of PSs at various concentrations in U87MG cells. The 700DX-PEG-cRGD, 700DX-PEG-PGlu-cRGD5, and 700DX-PEG-PGlu-cRGD15 revealed significantly high cellular uptake depending on the number of cRGD peptides, while no significant difference could be obtained between free 700DX and 700DX-PEG-PGlu-cRAD15. Especially at 700DX concentration of 200 nM, the fluorescence intensity of the cells treated by 700DX-PEG-PGlu-cRGD15 was 4.5 times and 2.5 times higher than that of cells incubated with free 700DX and 700DX-PEG-cRGD, respectively. In contrast, as shown in Fig. 2b, 700DX-PEG-cRGD, 700DX-PEG-PGlu-cRGD5, and 700DX-PEG-PGlu-cRGD15 did not show the drastic increase of cellular uptake in K562 cells. These results suggest the specificity of cRGD to α_v_β_3_ integrin and improved uptake by increasing the number of conjugated cRGD peptides. Note that free 700DX did not show concentration-dependent cellular uptake in both U87MG and K562 cells. Such low cellular uptake of 700DX was also reported in a different cell line in a previous study^26^, which is an inherent property of 700DX. To gain more insight about the specificity of 700DX-PEG-cRGD, 700DX-PEG-PGlu-cRGD5, and 700DX-PEG-PGlu-cRGD15 to α_v_β_3_ integrin, we also examined their cellular uptake in U87MG cells in the presence of excess free cRGDfK peptides (Supplementary Figure S17). The excess free cRGDfK peptides markedly inhibited the cellular uptake of the polymers containing cRGD peptides, and higher concentration of free cRGDfK peptides more efficiently lowered the cellular uptake, while 700DX and 700DX-PEG-PGlu-cRAD15 did not show the inhibited cellular uptake. Taken together, 700DX-PEG-cRGD, 700DX-PEG-PGlu-cRGD5, and 700DX-PEG-PGlu-cRGD15 should possess the selective affinity to α_v_β_3_ integrin.

To examine subcellular distribution of the PSs, we observed the U87MG cells incubated with the PSs, using confocal laser scanning microscopy (CLSM) (Fig. 2c). Consistent with the cellular uptake shown in Fig. 2a, 700DX-PEG-cRGD, 700DX-PEG-PGlu-cRGD5, and 700DX-PEG-PGlu-cRGD15 revealed considerably high fluorescence intensity of 700DX in the cells compared to free 700DX. Irrespective of cellular uptake efficiency, all the PSs were colocalized with lysotracker Red DND-99 that stains acidic organelles including lysosomes, suggesting that the PSs were internalized into the cells via an endocytic process.

### Cell viability assay

**Figure 3:**
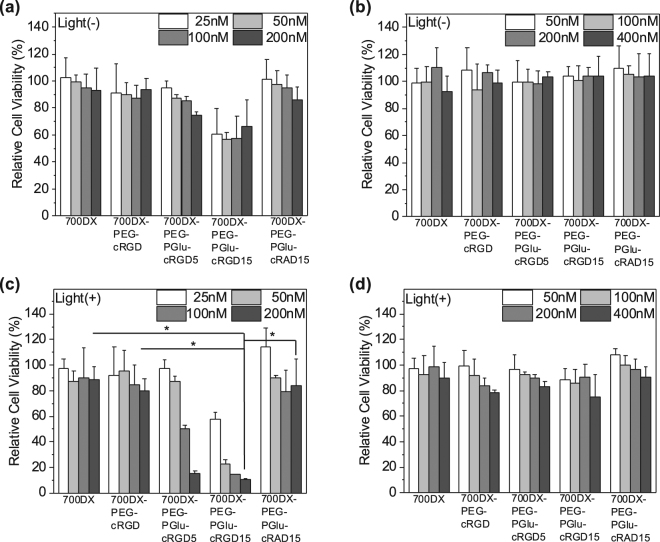
Dark toxicity and phototoxicity of PSs in (**a**,**c**) U87MG and (**b**,**d**) K562 cells. U87MG or K562 cells were incubated with PSs for 7 h. For phototoxicity, cells were washed with PBS thrice and exposed to light for 1 h in fresh medium. For dark toxicity, cells were washed with PBS thrice and incubated in fresh medium without photoirradiation. The results are expressed as mean ± S.D. (n = 6). Statistical analysis was performed using one-way ANOVA at 200 nM (*p < 0.01).

Successful PDT requires high phototoxicity and minimal dark toxicity. Cytotoxicity of the PSs in U87MG and K562 cells was evaluated by Cell Counting Kit-8 assay (Fig. 3). Free 700DX, 700DX-PEG-cRGD, and 700DX-PEG-PGlu-cRAD exhibited an ignorable dark toxicity in U87MG cells while 700DX-PEG-PGlu-cRGD5 showed slight cytotoxicity depending on concentration, and 700DX-PEG-PGlu-cRGD15 moderately reduced the cell viability (Fig. 3a). Meanwhile, all the PSs did not show dark toxicity in K562 cells (Fig. 3b). The dark toxicity of 700DX-PEG-PGlu-cRGD5 and 700DX-PEG-PGlu-cRGD15 in U87MG cells might be attributed to cell detachment and apoptosis induced by high concentration of cRGD peptides as previously reported^27^. As shown in Fig. 3c, for the U87MG cells, free 700DX and 700DX-PEG-PGlu-cRAD15 did not show phototoxicity, which is in line with the low cellular uptake in Fig. 2 and a previous study reporting negligible PDT effect of 700DX without antibodies^26^. It is noteworthy that 700DX-PEG-cRGD also exhibited little phototoxicity while it demonstrated the considerably enhanced cellular uptake (Fig. 2), suggesting that the cellular uptake offered by monomeric cRGD peptide might be insufficient to induce photochemical damage in this experimental condition. Consistent with these results, 700DX-PEG-PGlu-cRGD5 and 700DX-PEG-PGlu-cRGD15 at low concentration failed to induce PDT effect. However, they drastically exhibited phototoxicity at high concentration, where their cellular uptake was significantly increased. Interestingly, although cellular uptake of 700DX-PEG-PGlu-cRGD5 at 100 nM was similar with that of 700DX-PEG-cRGD at 200 nM, 700DX-PEG-PGlu-cRGD5 obviously excelled at phototoxicity. Considering their similar cellular uptake and subcellular localization, this enhanced phototoxicity might be explained by the combinatorial effect of PDT and the aforementioned cytotoxicity induced by cRGD peptides. Different from these results obtained in U87MG cells, phototoxicity could not be observed in K562 cells even at high PS concentration (Fig. 3d), which is also in good agreement with the cellular uptake as discussed above. These results indicate that multiple cRGD peptides-conjugated 700DX can induce strong toxicity only to the target cells without untoward damage to the non-target cells even under photoirradiation.

### Biodistribution

**Figure 4:**
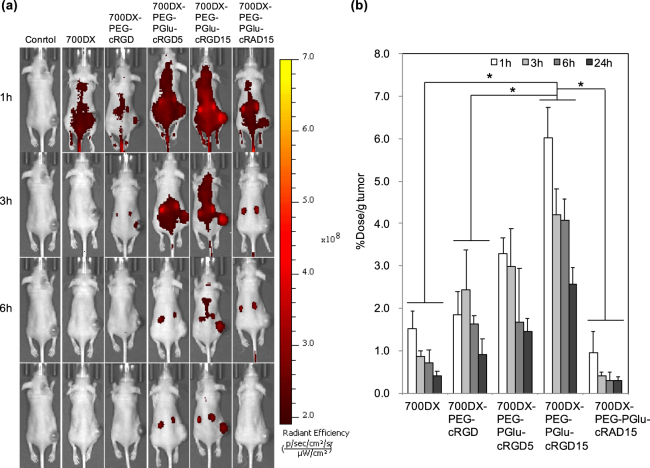
Biodistribution of PSs. (**a**) When the tumor size reached approximately 400 mm^3^, U87MG tumor-bearing BALB/c mice were intravenously injected with PSs (10 μg 700DX/mouse). IVIS images were obtained at 1, 3, 6 and 24 h after injection. (**b**) Biodistribution of PSs in tumor quantified by fluorescence intensity and expressed as a percentage of the injected dose/g tissue. The results are expressed as mean ± S.D. (n = 4). Statistical analysis was performed using two-way ANOVA with Holm-Sadic method (*p < 0.001).

To examine the biodistribution of the PSs, we observed localization of the intravenously injected PSs in mice bearing subcutaneous U87MG tumors using *in vivo* imaging system (IVIS) (Fig. 4a), and quantified the amount of the PSs in the tumors and normal organs by measuring the fluorescence intensity of PSs in their homogenized solutions (Fig. 4b and Supplementary Figure S18). Free 700DX and 700DX-PEG-PGlu-cRAD15 did not show tumor-specific accumulation and quickly disappeared from the body possibly through renal clearance as indicated by the high accumulation to the kidney (Supplementary Figure S18b). Although the tumor accumulation of 700DX-PEG-cRGD was slightly higher than free 700DX and 700DX-PEG-PGlu-cRAD15, the accumulation level was comparable to those in normal organs, which may be because of the insufficient affinity of monomeric cRGD peptide and eventual washout from the target site as reported by a previous study^28^. By contrast, 700DX-PEG-PGlu-cRGD5 and 700DX-PEG-PGlu-cRGD15 exhibited significantly enhanced tumor accumulation compared with the other PSs, and their accumulation level in the tumor was higher than those in the normal organs including liver, lung, spleen, muscle, and skin. Moreover, 700DX-PEG-PGlu-cRGD15 revealed high tumor accumulation level even 6 and 24 h after the injection. These results suggest the improved affinity to α_v_β_3_ integrin by multivalent effect^20^ and its usability to deliver 700DX to the target tumor. It should be noted that conjugation of multiple cRGD peptides to 700DX did not increase the accumulation level in the normal organs except for the kidney that should be the main elimination route through glomerular filtration, and the rapid renal clearance led to the quick disappearance of the PS from the blood (Supplementary Figure S18). The rapid clearance from the blood consequently increased tumor/blood accumulation ratio of the PS (Supplementary Figure S19), which is beneficial to avoid untoward photochemical damage to normal tissue including photosensitivity.

### Intratumoral distribution

**Figure 5:**
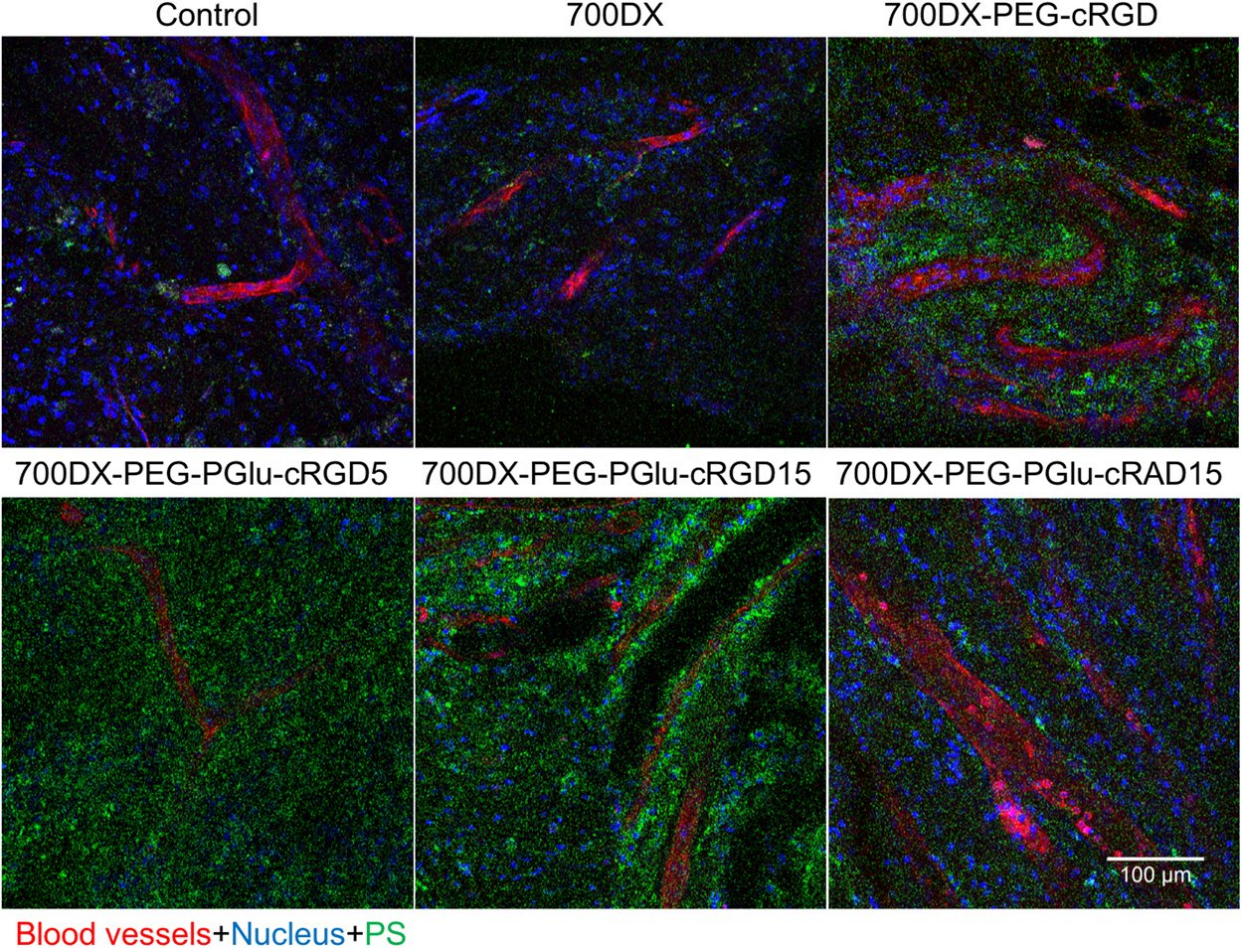
Representative CLSM images of intratumoral distribution of PSs. When the tumor size reached about 200 mm^3^, mice were intravenously injected with PSs (green). The nuclei and blood vessels were stained by Hoechst33342 (blue) and Lycopersicon esculentum lectin, DyLight 488 conjugate (red) respectively. The tumor was then directly observed by a CLSM immediately after collecting the tumor. The other fields of view are shown in Supplementary Figure S20.

Intratumoral distribution of the PSs 3 h after injection was investigated by observing tumor tissue by CLSM (Fig. 5 and Supplementary Figure S20). Consistent with tumor accumulation result (Fig. 4), the U87MG tumors treated with 700DX-PEG-PGlu-cRGD5 and 700DX-PEG-PGlu-cRGD15 exhibited appreciably higher fluorescence intensity than those treated with free 700DX and 700DX-PEG-PGlu-cRAD15. Monomeric cRGD also improved the accumulation of 700DX; however, compared with 700DX-PEG-PGlu-cRGD5 and 700DX-PEG-PGlu-cRGD15, its tumor accumulation was limited. These results again illustrate the higher tumor accumulation of the 700DX-PEG-PGlu-cRGD5 and 700DX-PEG-PGlu-cRGD15 via the enhanced avidities to αvβ3 integrin. In addition to the enhanced accumulation, these multiple cRGD-conjugated PSs revealed unique intratumoral distribution. Both of the 700DX-PEG-PGlu-cRGD5 and 700DX-PEG-PGlu-cRGD15 accumulated within tumor cells as well as tumor-associated vasculature, and 700DX-PEG-PGlu-cRGD15 was more preferentially localized on the vasculature without compromising penetration to a deep region. These results suggest that intratumoral distribution can be controlled by fine-tuning the number of cRGD peptides.

### *In vivo* antitumor activity

**Figure 6:**
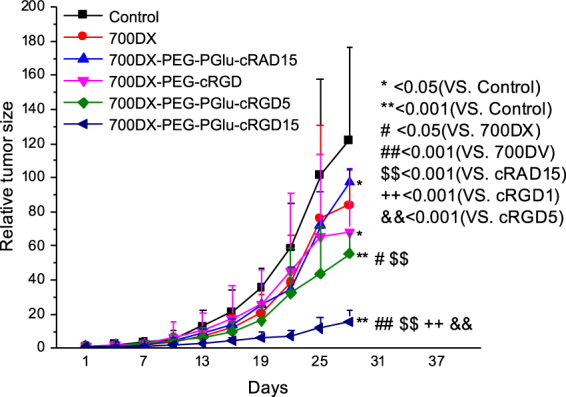
PDT efficacy of PSs. When the tumor size reached approximately 15 mm^3^, U87MG tumor-bearing BALB/c mice were intravenously injected with PSs (30 μg 700DX/mouse) on day 1, and the tumors were photoirradiated using a diode laser (100 J/cm^2^, 680 nm) 3 h after the injection. Tumor volumes were measured every three days until the tumor volume of the control reached 2000 mm^3^. Statistical analysis was performed using two-way ANOVA with Holm-Sidac method.

*In vivo* antitumor effect of PDT was examined in a subcutaneous U87MG tumor in a mouse (Fig. 6). U87MG tumors were treated with a single dose of photoirradiation [100 mW/cm^2^, 1000 sec] 3 h after intravenous administration of the PSs. It is obvious that all the PSs delayed the tumor growth; however, no statistical significance could be obtained among 700DX, 700DX-PEG-cRGD, and 700DX-PEG-cRAD15. These antitumor effects were consistent with the accumulation result (Fig. 4b), suggesting the direct photochemical damage to kill tumor cells was limited by the lower local concentration of PS in tumor (Fig. 5). Meanwhile, 700DX-PEG-PGlu-cRGD5 and 700DX-PEG-PGlu-cRGD15 significantly inhibited the tumor growth. These high antitumor effects can be partially explained by the high tumor accumulation (Fig. 4b). It is noteworthy that 700DX-PEG-PGlu-cRGD15 more remarkably inhibited the growth of tumors than 700DX-PEG-PGlu-cRGD5, although the tumor accumulation of 700DX-PEG-PGlu-cRGD5 and 700DX-PEG-PGlu-cRGD15 was similar 3 h after injection (Fig. 4b). Considering the more preferential accumulation of 700DX-PEG-PGlu-cRGD15 on the tumor-associated vasculature (Fig. 5), the appreciable difference in antitumor effects between 700DX-PEG-PGlu-cRGD5 and 700DX-PEG-PGlu-cRGD15 might be caused by tumor vasculature damage, which may also explain the difference between 700DX-PEG-PGlu-cRGD5 and 700DX-PEG-cRGD. These results suggest that spatial control of 700DX distribution in the tumor is important to improve PDT effect.

## Discussion

Since travel distance of singlet oxygen that is considered to be the most cytotoxic ROS in PDT is several ten nanometers in cells^29^, therapeutic outcome of PDT is governed by selective accumulation of PS in malignant cells. In this regard, 700DX-conjugated antibodies are powerful tools because they permit the selective delivery of 700DX to the target cell, and exhibits phototoxicity only when they are bound to the target cell and activated with a particular laser at the tumor site^4–10,12,13,26,30^. However, the antitumor efficacy of such PS delivery systems is sometimes compromised by their limited penetration from perivascular regions to the deep parts of the tumor. Previous studies reported that limited penetration of antibodies should be due to too strong binding of antibodies to cells peripheral to blood vessels, which phenomenon is so-called a binding site barrier^31^. Thus, fine-tuning affinity of delivery systems is important to control intratumoral distribution. In this context, we have utilized PEG-PGlu as a platform to mount 700DX and multiple cRGD peptides whose affinity could be controlled by changing the number of cRGDs. The increase in the number of cRGD peptides has been reported to permit multivalent binding, constant rebinding owing to the high local concentration of the peptide, and integrin clustering initiated by cRGD binding to the cell surface, thereby augmenting affinity, cellular uptake, tumor specificity, and tumor retention^32^. The increased cellular uptake and tumor accumulation were indeed obtained in many tetrameric cRGD peptides conjugate systems^17,19,33^. Our 700DX-PEG-PGlu-cRGD5 also exhibited the improved cellular uptake (Fig. 2) and tumor accumulation (Fig. 4), and, importantly, they were further improved by increasing the number of cRGD to 15. It should be noted that such improvement by conjugating more than 10 cRGDs to one molecule was rarely reported^34^ presumably because of difficulty in synthesis or poor hydrophilicity caused by the massive number of cRGD peptides. In this study, we utilized highly hydrophilic PEG-PGlu as a platform; more than 10 cRGD peptides could be conjugated to one construct, revealing that 15 cRGD peptides permit further improvement in tumor accumulation without binding site barrier (Fig. 5). Note that many previous studies developed PS delivery systems conjugated with multiple cRGD peptides and succeeded in enhanced PDT efficacy^35–37^. However, few studies demonstrated the effect of the number of cRGD peptides on tumor accumulation and microscopic intratumoral distribution of PS. Thus, the results obtained in this study should provide novel fundamental knowledge for this effect.

Although the cRGD modified polymer or nanoparticles showed high penetration in our and previous studies^21–23^, delivery to cells that are distal from functioning blood vessels may be still limited by irregular blood flow, the compression of blood vessels, increased interstitial fluid pressure, heterogeneous α_v_β_3_ integrin expression and structure of the extracellular matrix^38^. Also, a previous study reported moderate PDT effect of monomeric cRGD-conjugated PS irrespective of drastic enhancement in the tumor accumulation possibly due to the limited intratumoral oxygen level, demonstrating that the intratumoral concentration of PS should not always correlate with the ultimate therapeutic efficacy^39^. Thus, elimination of all the tumor cells is sometimes difficult only by directly giving oxidative damage to tumor cells in PDT. In this regard, many studies suggested that the tumor-associated vasculature targeted PDT could enhance the therapeutic efficacy^40^. By shutting down tumor-associated vasculature, cells surviving the direct oxidative damage should be killed by lack of oxygen and nutrition. However, conventional PSs do not have selectivity to tumor-associated vasculature. To conduct the tumor-associated vasculature targeted PDT, light should be delivered while blood contains high concentration of PS, which also damages blood vessels in normal tissues. To selectively damage tumor-associated vasculature, the PS should be delivered to tumor-associated vasculature and quickly cleared from blood. In our study, 700DX-PEG-PGlu-cRGD5 and 700DX-PEG-PGlu-cRGD15 could accumulate in tumor-associated vasculature (Fig. 5), while the PS in blood was quickly decreased within 3 h after injection (Supplementary Figure S18c). Because the molecular weight of our 700DX-polymer conjugates were smaller than 40 kDa (threshold of glomerular filtration)^41^, the rapid disappearance of our 700DX-polymer conjugates in blood should be mediated by renal clearance, as indicated by the high accumulation in the kidney (Supplementary Figure S18b). Thus, different from the tumor-associated vasculature with high accumulation of the PSs, the normal blood vessels accidentally exposed to photoirradiation are expected to avoid photochemical damage.

As mentioned above, our 700DX-PEG-PGlu-cRGD15 demonstrated efficient penetration in the tumor as well as preferential accumulation to the tumor vasculature, thereby accomplishing efficient antitumor effect. Unlike our study, in some other research about multiple cRGD-conjugated systems^42,43^, cRGD conjugated nanoparticles could accumulate only in edge part or near-vessel part of tumor. The limited penetration of these materials was ascribed to their large size, as reducing the size of cRGD-conjugated nanomaterials could improve the penetration ability^43^. Thus, the small size of the conjugates in the present study might contribute to the high penetration. Another reason of the efficient penetration might be a possible transcytosis pathway through cRGD-integrin mediated active transport. This hypothesis was used to explain high penetration of cRGD-conjugated micelles in 3D U87MG glioma spheroids in a previous research^23^. The micelle covered by cRGDs demonstrated superior efficacy to penetrate into the deeper part of 3D U87MG glioma spheroids compared to similar micelle without cRGD peptide modification. Similar improvement was also observed in other micelle^21^ and nanoparticle^22^.

In summary, we developed the 700DX-polymer conjugates containing multiple cRGDs that could show a high affinity to tumor cells overexpressing α_v_β_3_ integrin. These conjugates exhibited enhanced cellular uptake in the cultured cells by strong interaction with α_v_β_3_ integrin and achieved targeting delivery and prolonged retention at tumor site with controlled intratumoral distribution. Owing to these improvements, the 700DX-polymer conjugates significantly inhibited the growth of tumor. Thus, the multiple cRGD peptides conjugated polymer is a promising structure for PDT of 700DX.

## Methods

### Materials

1,1′-Carbonyldiimidazole (99%) and penicillin-streptomycin were obtained from Sigma-Aldrich Chemical Co. (St. Louis, MO). IRDye® 700DX NHS Ester was obtained from LI-COR, Inc. (Lincoln, NE). Cyclic RGDfK peptide and cyclic RADfK peptide were purchased from Synpeptide (Shanghai, China). γ-Benzyl-L-glutamate *N*-carboxyanhydride was purchased from Chuo Kaseihin Co., Inc. (Tokyo, Japan). Azide functionalized PEG having a terminated hydroxyl group (azide-PEG-OH) was obtained from NOF Corporation (Tokyo, Japan). DBCO-NHS was purchased from Click Chemistry Tools LLC. (Scottsdale, AZ). Lysotracker Red DND-99 and Hoechst33342 were obtained from Thermo Fisher Scientific, Inc. (Waltham, MA). Methanesulfonyl chloride (MsCl) and 2,4,6-trinitrobenzenesulfonic acid hydrate (TNBS) were purchased from Tokyo Chemical Industry Co., Ltd. (Tokyo, Japan). Dimethyl sulfoxide (DMSO), *n*-hexane, benzene, triethylamine (TEA), tetrahydrofuran (THF) and sodium hydroxide solution were purchased from Wako Pure Chemical Industries Ltd. (Osaka, Japan). Methanol was purchased from Kanto Chemical Co., Inc. (Tokyo, Japan). DMSO, THF, TEA, and MsCl were distilled over CaH_2_ prior to use. Other solvents were directly used without treatment. A series of dialysis membrane with different MWCO were purchased from Spectrum Laboratories, Inc. (Rancho Dominguez, CA) and the centrifugal filter was purchased from Merck Millipore Ltd. (Billerica, MA).

### Synthesis of azide-PEG-NH_2_

To transform the terminative hydroxyl groups of azide-PEG-OH (M_n_ ~12,000) to an amino group, azide-PEG-OH (2.4 g, 0.2 mmol) was dissolved in 20 mL of benzene, and freeze-dried. The dried azide-PEG-OH was dissolved in 20 mL of anhydrous THF with 150 μL of TEA (1.0 mmol, 5.0 equiv) under argon atmosphere. Four milliliters of THF solution containing 50 μL of MsCl (0.7 mmol, 3.5 equiv) was then added dropwise into the azide-PEG-OH solution. The reaction protected against exposure to light was allowed to proceed at room temperature for 12 h under argon atmosphere. The reaction solution was added dropwise into 700 mL of *n*-hexane to obtain precipitation. The precipitate was collected by vacuum filtration and vacuum drying to produce about 2.1 g of azide functionalized PEG having a terminated methanesulfonyl group (azide-PEG-mesyl). Azide-PEG-mesyl was then dissolved in 160 mL of aqueous ammonia at the concentration of 25% (w/w). The reaction protected against exposure to light was allowed to proceed at room temperature for 4 days. After removing the excess ammonia by a rotary evaporator, the solution was dialyzed against deionized water thrice using dialysis membrane (MWCO, 6,000–8,000 Da) at room temperature for 24 h. The crude product was purified by ion exchange column, CX-Sephadex C-50 (Sigma-Aldrich Chemical Co.). The drenched column was first washed with 0.5% CH_3_COOH solution until pH of the solution unchanged after passing the column. Then the acid in the column was washed with deionized water. After the preparation of column, the polymer solution was added dropwise into the column, and then the column was washed with deionized water until the eluent became negative for the indicator for PEG, ammonium thiocyanate solution (0.174 g/ml). Then, 0.125% NH_3_, diluted from 25% (w/w) ammonia, was added into the column to elute the azide-PEG-NH_2_. After removal of ammonia using a rotary evaporator, the solution was dialyzed against deionized water at room temperature for 12 h using dialysis membrane (MWCO, 6,000–8,000), and the final product was obtained by freeze-drying (1.36 g).

### Synthesis of azide-PEG-poly(γ-benzyl-L- glutamine) (PEG-PBLG)

Azide-PEG-NH_2_ (1.2 g, 0.1 mmol) was dissolved in 20 mL of benzene. After freeze-drying, the azide-PEG-NH_2_ was dissolved in 20 mL of anhydrous DMSO. Ten milliliters of DMSO solution containing 1.26 g of BLG-NCA (4.8 mmol, 48 equiv) was added into the PEG solution under argon atmosphere. The reaction was allowed to proceed at 40 °C for 3 days under argon atmosphere. The reaction solution was added dropwise into 600 mL of diethyl ether to obtain precipitation of PEG-PBLG. The PEG-PBLG was collected by vacuum-filtration and subsequent vacuum-drying (1.91 g). The polymerization degree of PBLG in PEG-PBLG was determined to be 40 by ^1^H NMR. Mw/Mn was determined to be 1.10 by GPC [column: TSK-gel superAW3000, superAW4000, and superAWL-guard column (Tosoh Corporation, Yamaguchi, Japan); eluent: NMP containing 50 mM LiBr; flow rate: 0.3 ml/min; detector: refractive index (RI); temperature: 40 °C].

### Acetylation of PEG-PBLG

To acetylate the *ω*-end amino group in PEG-PBLG, 1.1 g PEG-PBLG was dissolved in 10 mL anhydrous DMSO with excess TEA. After 1 h stirring at room temperature, excess acetic anhydride was added to the solution. After overnight stirring, the solution was dialyzed against methanol twice and deionized water twice with dialysis membrane (MWCO, 12,000–14,000 Da) at room temperature for 24 h. The acetylated PEG-PBLG (azide-PEG-PBLG-acetic) was obtained by freeze-drying (1.02 g).

### Synthesis of azide-PEG-poly(L-glutamic acid) (PEG-PGlu)

To deprotect benzyl groups in the PBLG segment of azide-PEG-PBLG-acetic, 1.0 g of azide-PEG-PBLG-acetic was added in 20 mL of 0.5 M NaOH solution. After 12 h stirring, the solution was dialyzed against 0.01 M HCl and deionized water using dialysis membrane (MWCO, 12,000–14,000 Da) at room temperature, followed by freeze dry to obtain 0.85 g of the product.

### Synthesis of cRGD-functionalized PEG-PGlu (azide-PEG-P[Glu/Glu(cRGD)])

To activate the carboxyl groups of PEG-PGlu, PEG-PGlu (20 mg, 1.17 μmol) and CDI (15.1 mg 93.6 μmol) were dissolved in 2 mL of anhydrous DMSO. After 2 h reaction, 2 mL DMSO solution containing cRGDfK (146 mg, 243 μmol) and TEA was added dropwise into the above CDI-activated PEG-PGlu solution. The reaction mixture was stirred at room temperature overnight to produce azide-PEG-P[Glu/Glu(cRGD)]. The mixture was dialyzed against deionized water at room temperature using dialysis membrane (MWCO, 12,000–14,000 Da), and the final product containing 15 cRGDfK peptides, azide-PEG-P[Glu/Glu(cRGD)_15_] was obtained by freeze-drying (24 mg). The other cRGDfK conjugated polymer containing 5 cRGDfK peptides, azide-PEG-P[Glu/Glu(cRGD)_5_] was produced by changing the ratio of CDI to PEG-PGlu to 40 and ratio of cRGDfK to PEG-PGlu to 80. Azide-PEG-P[Glu/Glu(cRAD)_15_] was synthesized in the similar method. Then the polymer was purified by HPLC Fraction [column: Superdex200 increased 10/300 GL (GE Healthcare, Chicago, IL); eluent: 10 mM PBS buffer containing 140 mM NaCl; flow rate: 0.5 ml/min]. The number of conjugated cRGDfK peptides or cRADfK peptides was determined by ^1^H NMR.

### Synthesis of 700DX-PEG-PGlu-cRGDx

To convert the terminal azide group to primary amine group, azide-PEG-P[Glu/Glu(cRGD)] was reacted with DTT (100 equiv) in 2 mL of 0.1 M NaHCO_3_ buffer (pH 8.5) at room temperature overnight. After dialysis against deionized water and lyophilization, the conversion rate of azide group to amine group was determined to be 90.3% by TNBS Method. The polymer was then dissolved in DMSO, and mixed with DMSO containing 700DX-NHS (1.2 equiv) to conjugate 700 DX to the primary amine group. After dialysis against deionized water at room temperature, the solution was concentrated by a centrifugal filter (MWCO, 10,000), and passed through the Disposable PD-10 Desalting Column (GE Healthcare) to remove unreacted 700DX. The product was obtained by lyophilization and further purified by HPLC. In the aforementioned procedures in which 700DX was included, light was avoided as much as possible. The obtained polymer was characterized by GPC.

### Synthesis of 700DX-PEG-cRGD (700DX -PEG-cRGD)

cRGDfK (6 mg) was dissolved in 1 mL of DMSO, and mixed with DMSO containing DBCO-NHS (0.2 equiv) to conjugate cRGDfK to the azide group of azide-PEG-NH_2_. After 12 h reaction at room temperature, 4 mL deionized water was added into the solution to break unreacted NHS groups. After another 24 h, 12 mg of azide-PEG-NH_2_ was added into the solution. To promote the click-reaction, the solution was frozen and thawed^44^. After freeze-thawing three times, the solution was dialyzed (MWCO, 6,000–8,000 Da) against deionized water at room temperature. The product was obtained by lyophilization. The introduction of cRGD to polymer was confirmed by ^1^H NMR. The obtained polymer was dissolved in DMSO, and mixed with DMSO containing 700DX-NHS (1.2 equiv) to conjugate 700 DX to the amine group. After dialysis (MWCO, 6000 ~ 8000 Da) in deionized water 3 times, the solution was concentrated by a centrifugal filter (MWCO, 10,000) and passed through the Disposable PD-10 Desalting Column to purify the polymer. In the aforementioned procedures in which 700DX was included, light was avoided as much as possible. The product was obtained by lyophilization and further purified by HPLC.

### Fluorescence measurement

Fluorescence spectra of 700DX and the 700DX-conjugated polymers in water (200 nM of 700DX) were measured using a fluorophotometer (FP-8300, JASCO Corporation, Tokyo, Japan). Excitation wavelength was 550 nm.

### Cell Culture

U87MG and K562 cell lines were purchased from American Type Culture Collection (ATCC) (Manassas, VA). U87MG cells were grown in Eagle’s minimum essential medium containing 10% fetal bovine serum and 1% penicillin and streptomycin. The medium for K562 was Iscove’s modified dulbecco’s medium containing 10% FBS and 1% penicillin and streptomycin. Cells were incubated at 37 °C in humidified atmosphere containing 5% CO_2_.

### Cellular uptake

Cells were seeded in a 96-well plate (Greiner Bio-One GmbH, (Frickenhausen, Germany)) at a density of 1 × 10^4^ cells/well for 24 h. The culture medium was replaced with fresh medium containing the polymers at various concentrations in the absence or presence of free cRGDfK peptides. After 7 h incubation and medium replacement, the cells were lysed by 100 μL of Passive Lysis Buffer (Promega Corporation, (Madison, WI)). After 30 min incubation, 50 μL of lysate from each well was transferred to black 96 well plates (Thermo Fisher Scientific, Inc. (Waltham, MA)), and the fluorescence of 700DX was quantified by *in vivo* imaging system (IVIS, Perkin Elmer, Waltham, MA) (ex/em = 710 nm/780 nm). For each sample, the average fluorescence was calculated from 6 wells.

### Confocal laser scanning microscopic observation of subcellular distribution

U87MG cells were seeded at density of 1 × 10^5^ cells/dish in 35-mm glass-based dishes. After 24 h incubation, cells were incubated in fresh medium containing various concentrations of PSs for 7 h. To stain lysosomes, Lysotracker Red DND-99 was added into each well at concentration of 50 nM 30 min before the end of incubation. Cells were then washed three times by 1 ml of PBS and fixed by PBS containing 4% paraformaldehyde and Hoechst33342 (10 µg/mL) to stain cell nuclei. Cells were washed with PBS three times and then observed in PBS using a confocal laser scanning microscope (LSM710, Carl Zeiss AG, Oberkochen, Germany).

### Cell viability

The cytotoxicity was evaluated using the Cell Counting Kit 8 assay in U87MG cells and K562 cells. U87MG Cells were seeded in a 96-well plate at a density of 1 × 10^4^ cells/well for 24 h. For K562 cells, a U-shape 96-well plate (Greiner Bio-One GmbH) was used. The culture medium was replaced with fresh medium containing the PSs at various concentrations. After 7 h incubation and medium replacement, the cells were photoirradiated using a halogen lamp equipped with a band filter (400–700 nm) at fluence of 3.0 mW/cm^2^ for 1 h in fresh medium on ice, followed by additional incubation for 48 h. After replacing the old medium, 100 μL of medium containing 10 μL of Cell Counting Kit 8 solution was added to each well and cells were incubated for 2 h. The absorbance at a wavelength of 450 nm was measured using iMark Microplate Reader (BIO-RAD (Hercules, CA)). The cell viability was calculated from the following equation:

$${\rm{Cell}}\,{\rm{viability}}( \% )=([A]test-[A]blank)/([A]control-[A]blank)\times 100,$$

where [A]test, [A]control and [A]blank are the absorbance values of the cells treated with the samples, non-treated cells, and medium without cells, respectively. For each sample, the average absorbance was calculated from 6 wells.

### Biodistribution study

All animal experiments were approved by the Animal Care and Use Committee of Tokyo Institute of Technology, and performed in accordance with the Guidelines for the Care and Use of Laboratory Animals as stated by Tokyo Institute of Technology. Four-week-old female BALB/c nude mice were obtained from Charles River Laboratories Japan, Inc. (Yokohama, Japan). To make a subcutaneous tumor model, 1 × 10^6^ U87MG cells were injected subcutaneously in the left dorsum of each mouse. When the tumor volume reached approximately 400 mm^3^, PSs were intravenously injected to the mice (10 μg of 700DX/mouse), and fluorescence of 700DX in the body was imaged at indicated time points using IVIS (ex/em = 710 nm/780 nm). Also, to quantify the accumulated amount of 700DX, tumors and organs were taken out from the mice and gently washed by PBS at indicated time points. After removing the excess fluid by tissue paper, the tumors and organs were mixed with Passive Lysis Buffer, and homogenized by a Handy Sonic (TOMY SEIKO Co., Ltd., Tokyo, Japan). The homogenized solutions were transferred to black 96 well plates, and the fluorescence of 700DX was quantified using IVIS (ex/em = 710 nm/780 nm).

### Microscopic observation of intratumoral distribution

To observe intratumoral distribution of the PSs in tumor tissue, subcutaneous tumor models were prepared in the same way with the biodistribution study. When the tumor volume reached about 200 mm^3^, mice were injected with samples (10 μg of 700DX/mouse). Two and half hours after intravenous injection of the samples, the mice were additionally injected with 100 μL of PBS buffer containing 250 μg of Hoechst33342 and 25 μg of Lycopersicon esculentum lectin, DyLight 488 Conjugate (Vector Laboratories,(Burlingame, CA)) to stain nuclei and blood vessels, respectively. The tumor was then gently taken out and cut in half after sacrificing the mice. The tumor was directly observed by an LSM710 confocal laser scanning microscope, immediately after collecting the tumor.

### *In vivo* antitumor activity

To determine tumor volume, long axis (length) and short axis (width) from each tumor were measured using a caliper. Each tumor volume was calculated using the following equation:

$${\rm{tumor}}\,{\rm{volume}}={\rm{length}}\times {{\rm{width}}}^{2}\times 0.5.$$

Subcutaneous tumor models were prepared in the same way with the biodistribution study. Animals were randomized into 6 groups (6 mice/group): (1) no treatment, (2) 700DX, (3) 700DX-PEG-cRGD, (4) 700DX-PEG-PGlu-cRGD5, (5) 700DX-PEG-PGlu-cRGD15, and (6) 700DX-PEG-PGlu-cRAD15. When tumor volume reached approximately 15 mm^3^, the samples (30 μg of 700DX/mouse) were intravenously injected to the mice through tail veins. Three hours after intravenous injection, tumors were photoirradiated using a diode laser (680 nm) at fluence of 100 mW/cm^2^ for 1,000 s. Tumor volume was measured every three days until it reached 2,000 mm^3^.

### Statistical analysis

Statistical analysis was performed using one-way and two-way ANOVA with multiple comparison tests by graphpad prism 6.

## Electronic supplementary material

Supplementary Information
