# Supplementary material for: Effect of multiple cyclic RGD peptides on tumor accumulation and intratumoral distribution of IRDye 700DX-conjugated polymers
Source: Sci Rep. 2018 May 25;8:8126. doi: 10.1038/s41598-018-26593-0 (PMC5970177; doi:10.1038/s41598-018-26593-0)
Supplement: Supplementary file 1 — Supplementary Information [file 41598_2018_26593_MOESM1_ESM.pdf]

Supplementary Information

Effect of multiple cyclic RGD peptides on tumor accumulation and intratumoral distribution of IRDye 700DX-conjugated polymers

Xuebo Dou[1], Takahiro Nomoto[1,*], Hiroyasu Takemoto[1], Makoto Matsui[1], Keishiro Tomoda[1] and Nobuhiro Nishiyama[1,2,*]

[1]Laboratory for Chemistry and Life Science, Institute of Innovative Research, Tokyo Institute of Technology, 4259 Nagatsuta-cho, Midori-ku, Yokohama, Kanagawa, 226-8503, Japan

[2]Innovation Center of Nanomedicine (iCONM), Kawasaki Institute of Industrial Promotion, 3-25-14 Tonomachi, Kawasaki-ku, Kawasaki, Kanagawa, 210-0821, Japan

* nomoto@res.titech.ac.jp

* nishiyama.n.ad@m.titech.ac.jp

Supplementary Figures

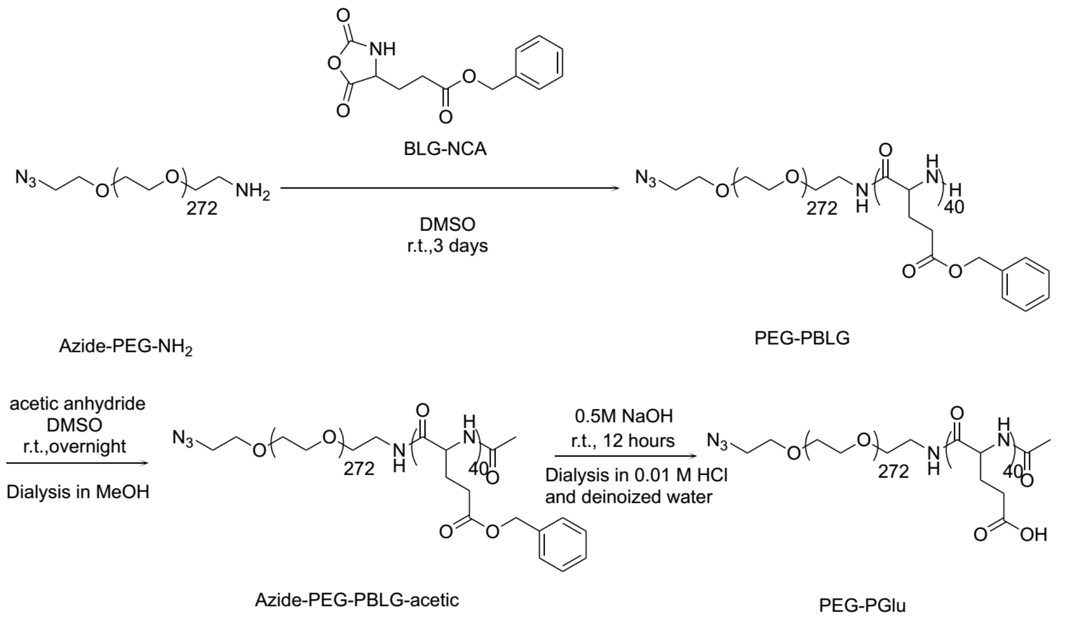

Supplementary Figure S1. Synthesis of PEG-poly(L-glutamic acid) (PEG-PGlu).

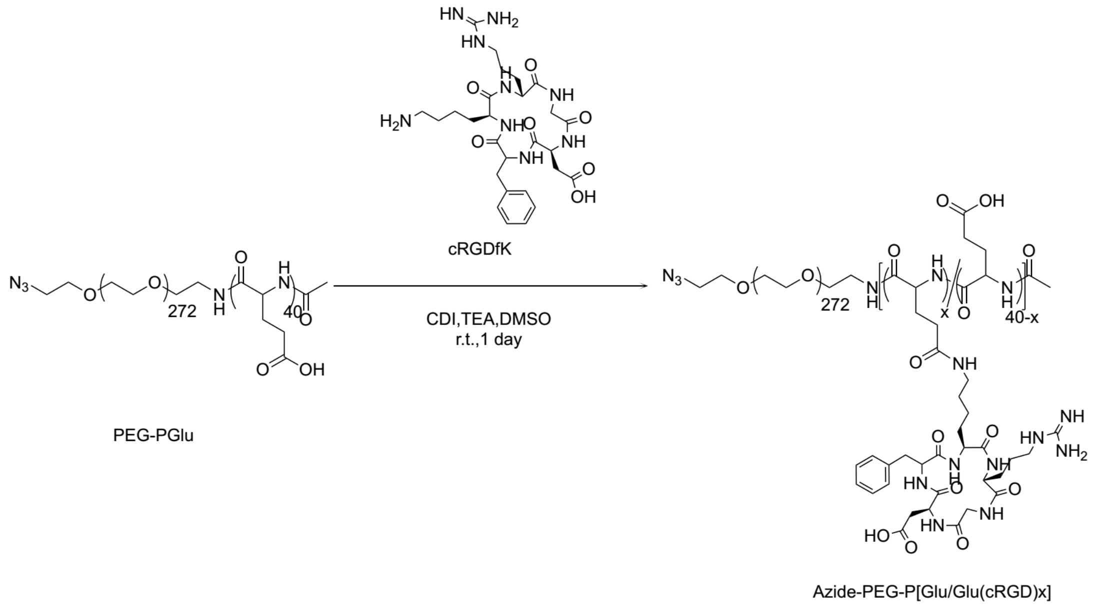

Supplementary Figure S2. Synthesis of Azide-PEG-P[Glu/Glu(cRGDx)].

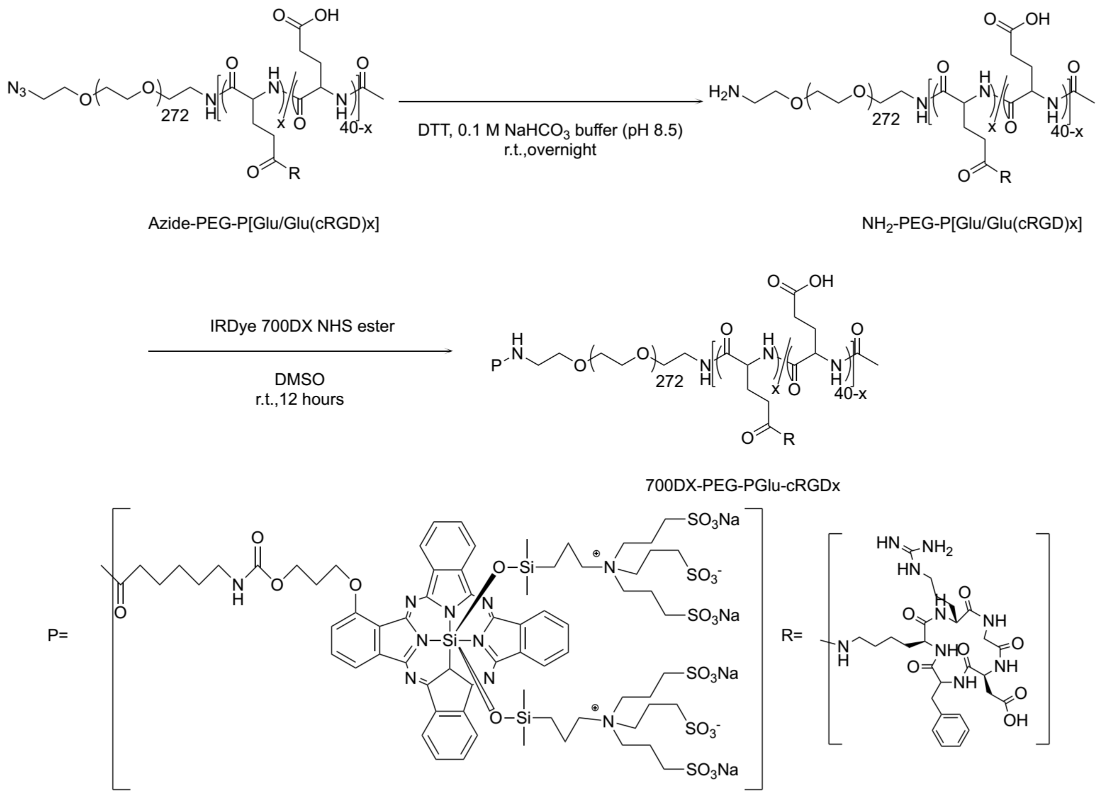

Supplementary Figure S3. Synthesis of 700DX-PEG-PGlu-cRGDx.

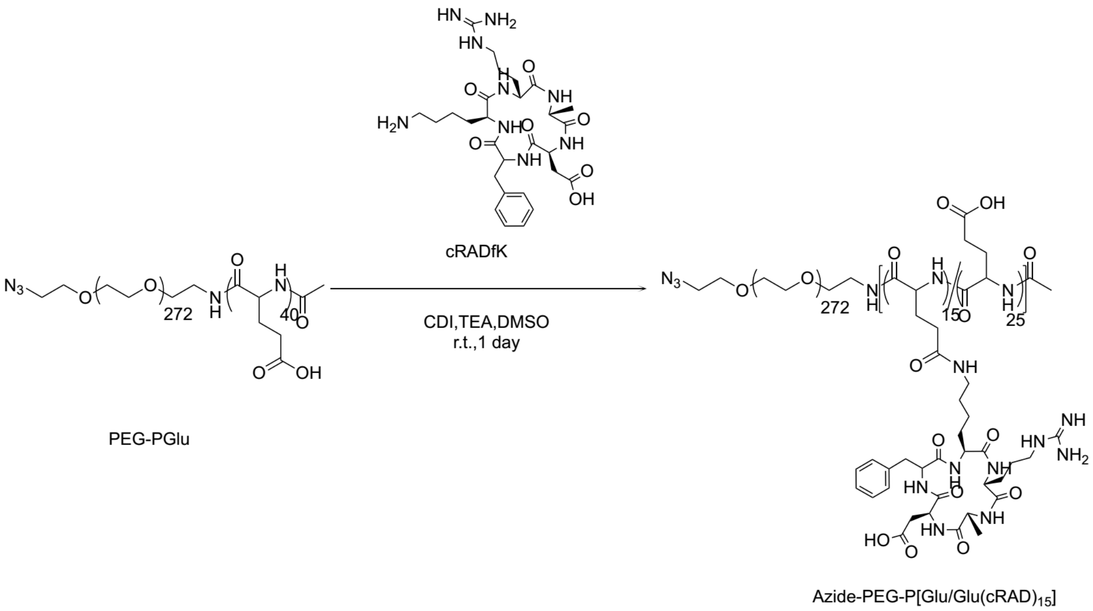

Supplementary Figure S4. Synthesis of Azide-PEG-P[Glu/Glu(cRAD)$_{15}$].

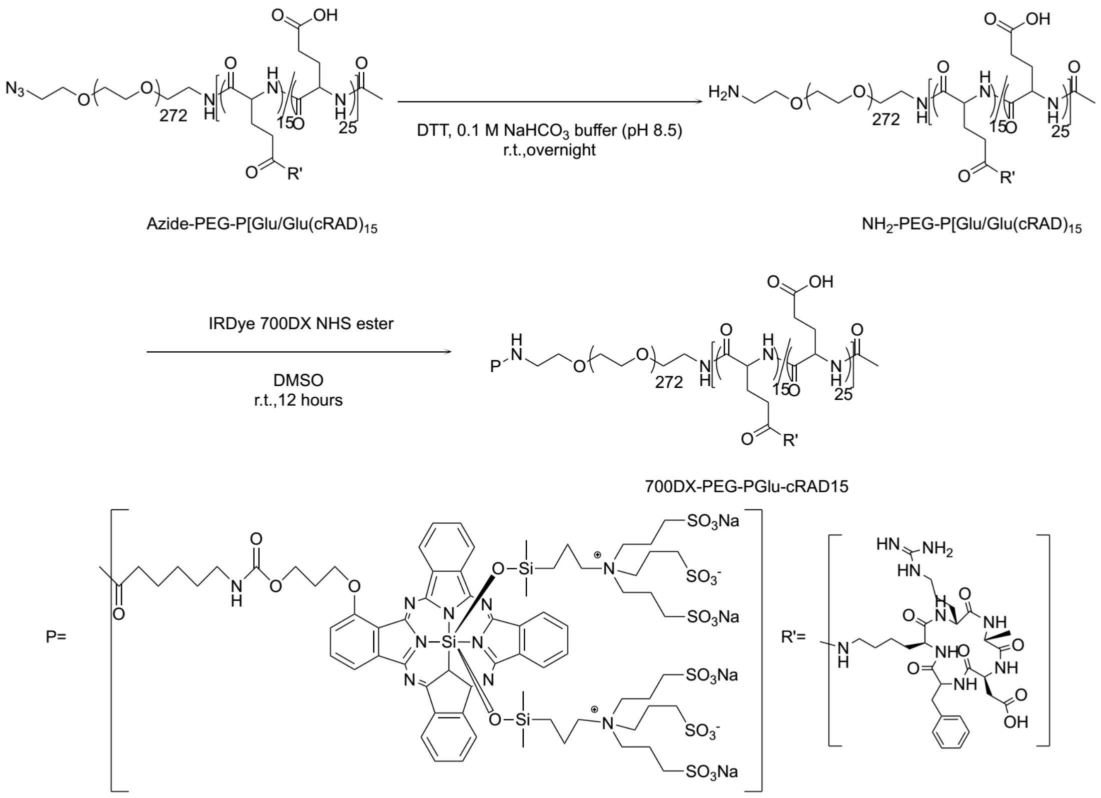

Supplementary Figure S5. Synthesis of 700DX-PEG-PGlu-cRAD15.

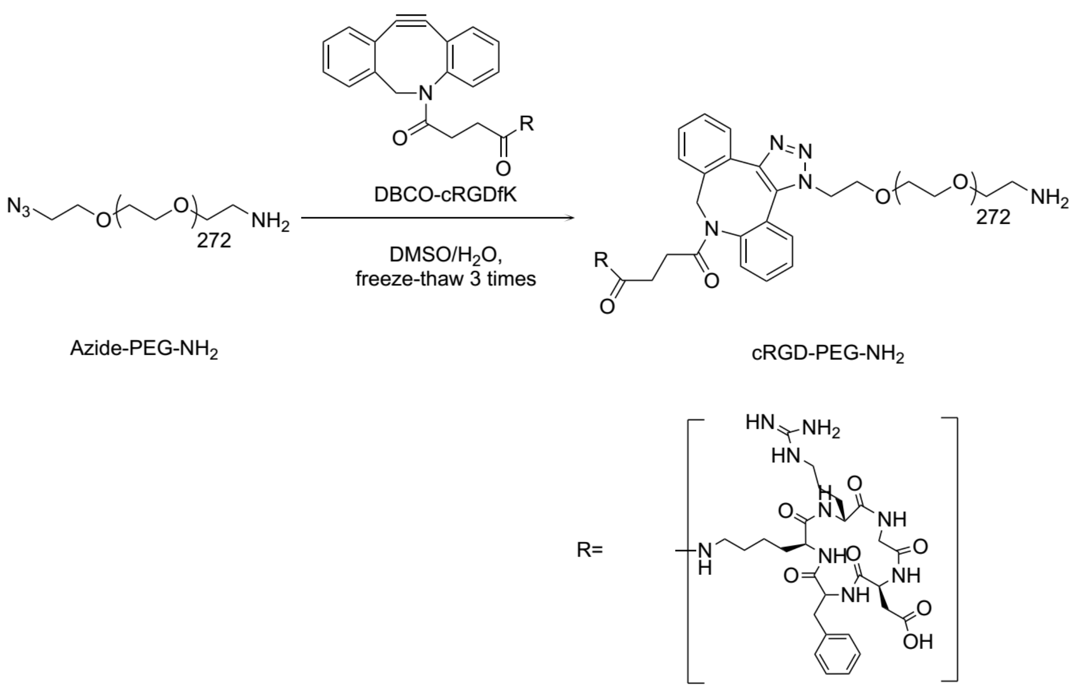

Supplementary Figure S6. Synthesis of cRGD-PEG-NH_2.

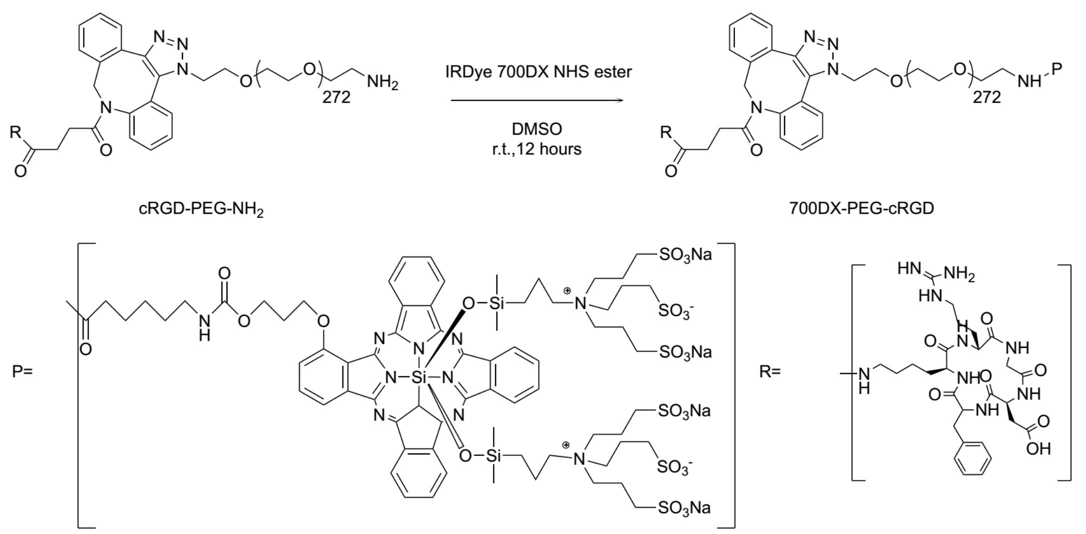

Supplementary Figure S7. Synthesis of 700DX-PEG-cRGD.

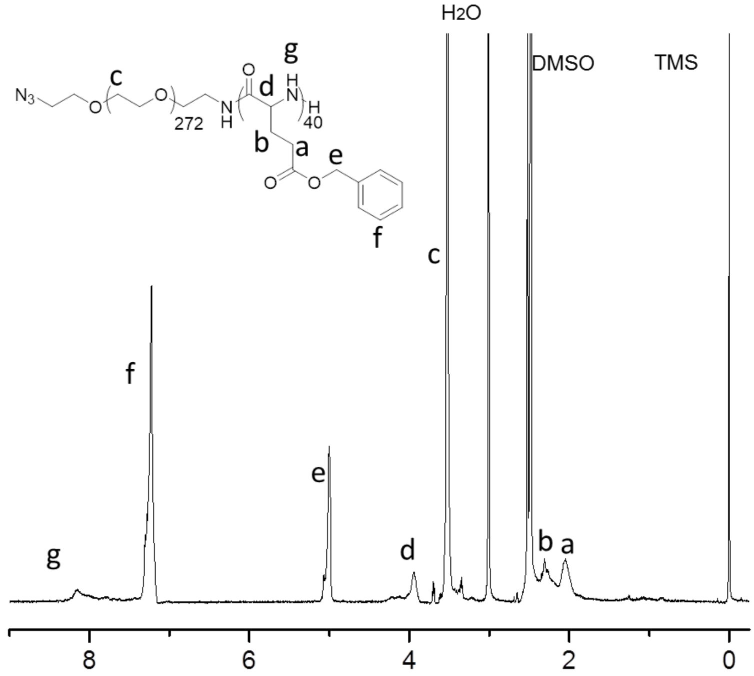

Supplementary Figure S8. ^{1}H NMR spectrum of azide-PEG-PBLG (DMSO-d_6, 80°C).

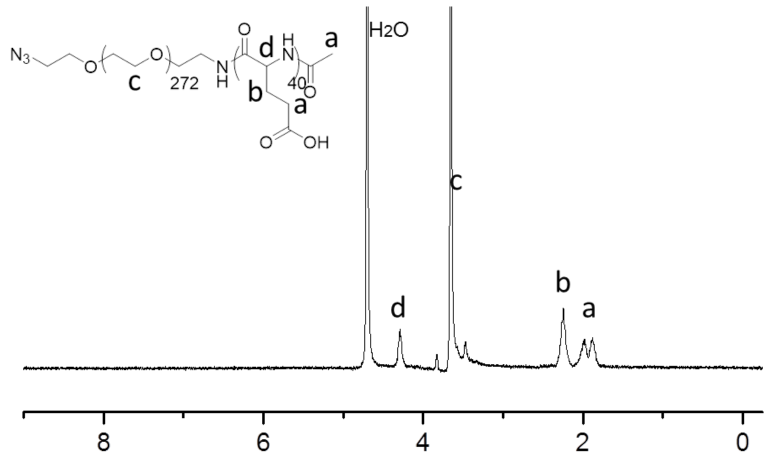

Supplementary Figure S9. ^1H NMR spectrum of azide-PEG-PGlu (D_2O, 25°C).

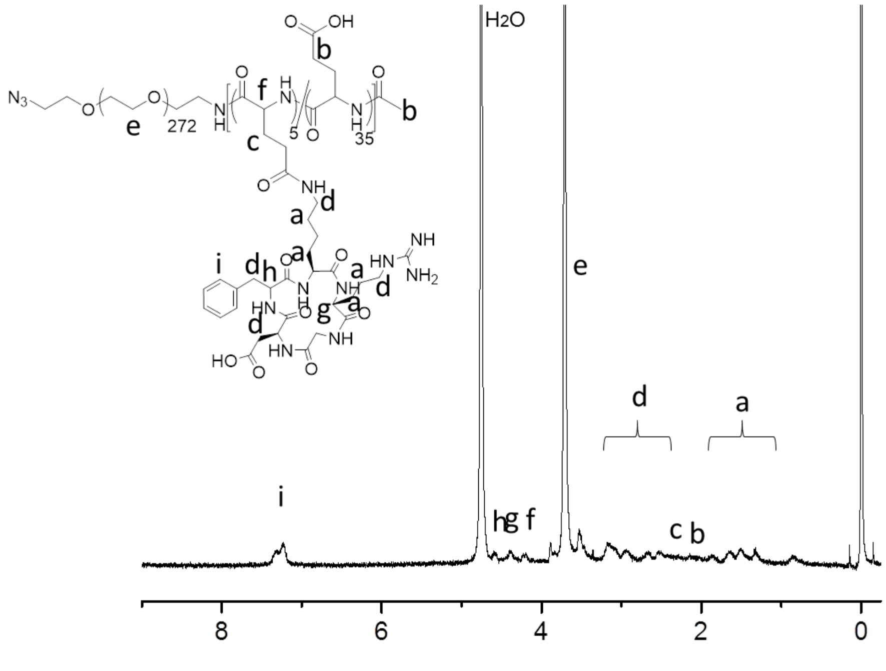

Supplementary Figure S10. ^1H NMR spectrum of azide-PEG-PGlu-cRGD5 (D_2O, 25°C).

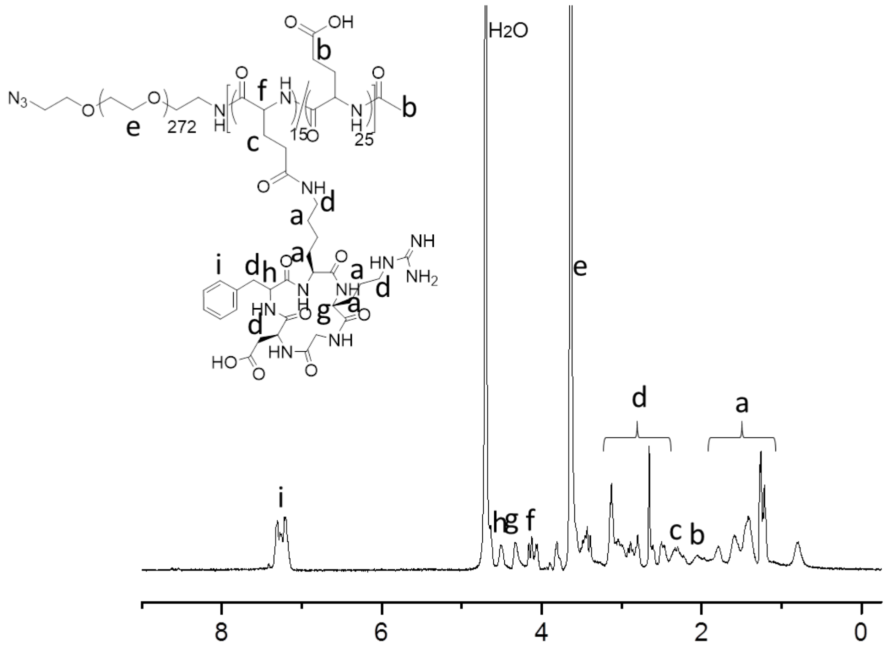

Supplementary Figure S11. ^{1}H NMR spectrum of azide-PEG-PGlu-cRGD15 (D_2O, 25°C).

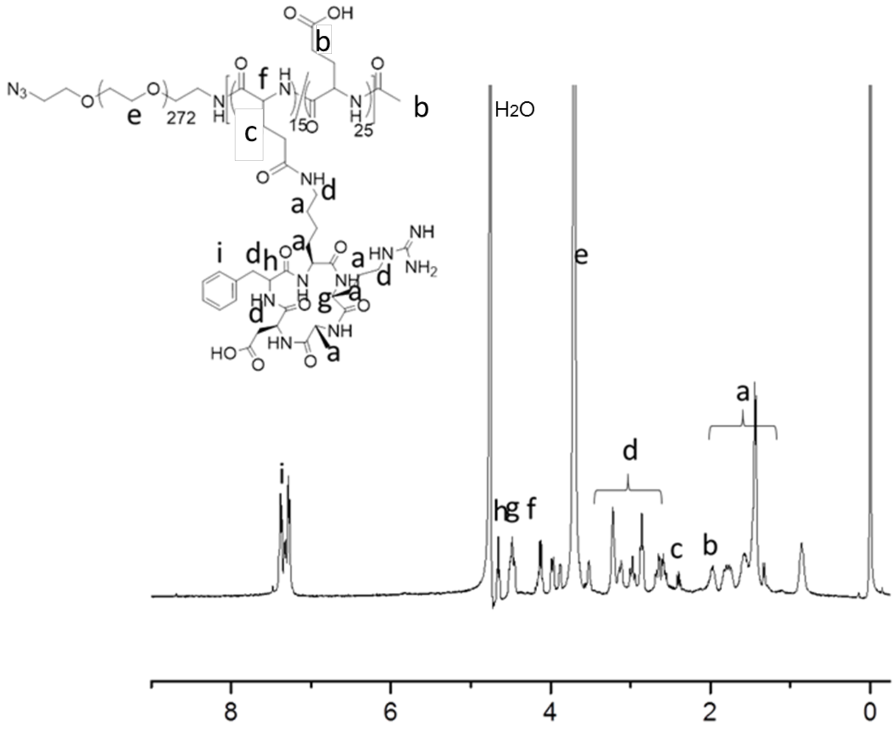

Supplementary Figure S12. ^{1}H NMR spectrum of azide-PEG-PGlu-cRAD15 (D_2O, 25°C).

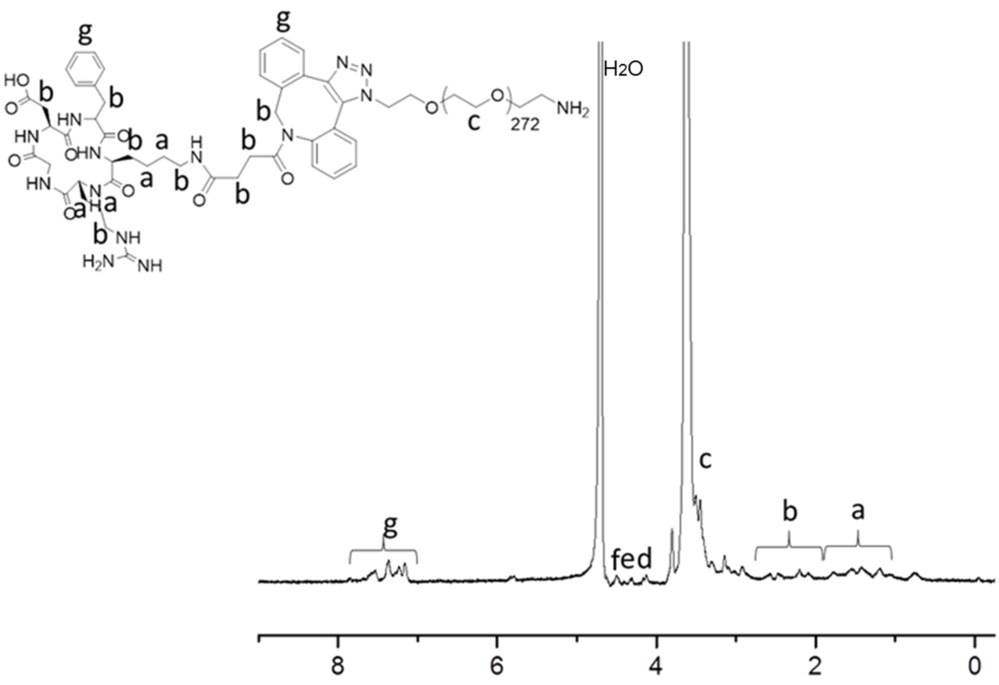

Supplementary Figure S13. ^{1}H NMR spectrum of cRGD-PEG-NH_2 (D_2O, 25°C).

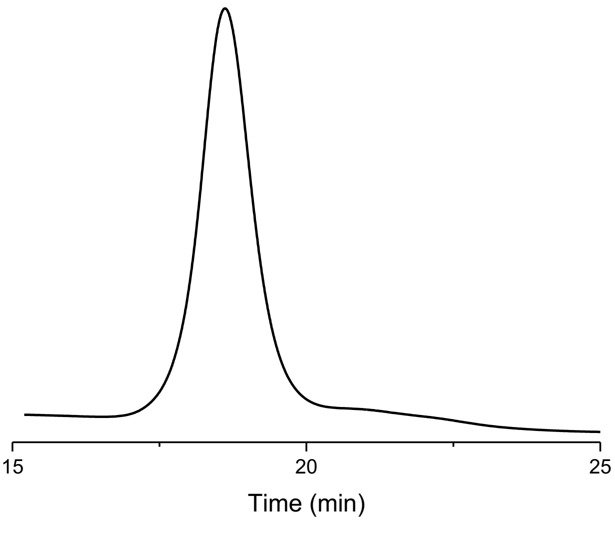

Supplementary Figure S14. GPC result of PEG-PBLG. [Column: TSK-gel superAW3000, superAW4000, and superAWL-guard column (Tosoh Corporation, Yamaguchi, Japan); eluent: NMP containing 50 mM LiBr; flow rate: 0.3 ml/min; detector: refractive index (RI); temperature: 40 °C]

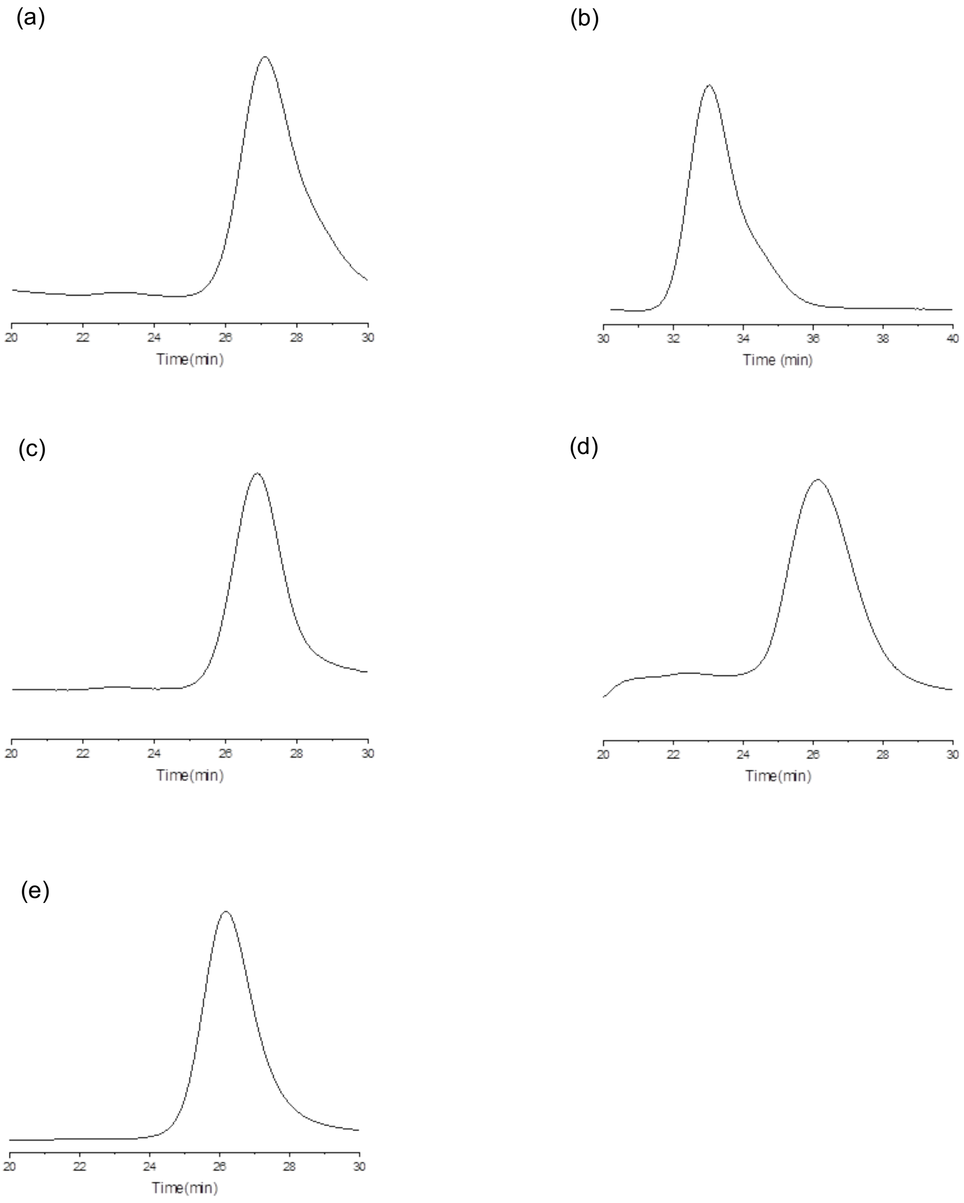

Supplementary Figure S15. GPC results of (a) PEG-PGlu, (b) 700DX-PEG-cRGD, (c) 700DX-PEG-PGlu-cRGD5, (d) 700DX-PEG-PGlu-cRGD15, and (e) 700DX-PEG-PGlu-cRAD15. [Column: Superdex 200 increase 10/300 GL (GE Healthcare Life Sciences, Marlborough, MA); eluent: 10 mM PBS buffer containing 140 mM NaCl; flow rate: 0.5 ml/min, detector: ultraviolet (UV, wavelength: 220nm)]

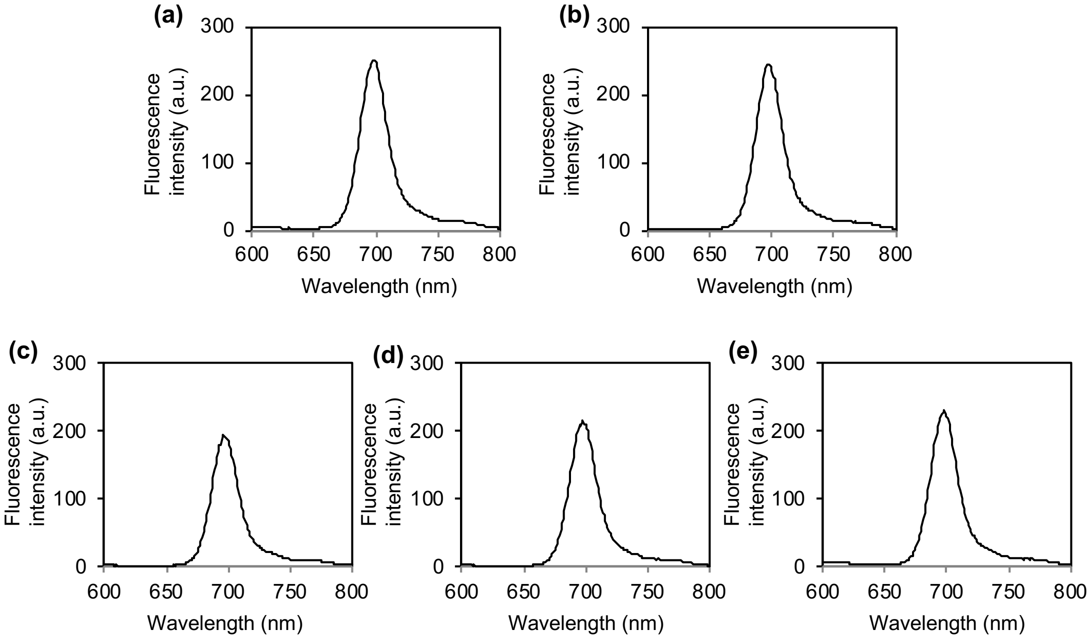

Supplementary Figure S16. Fluorescence spectra of (a) 700DX, (b) 700DX-PEG-cRGD, (c) 700DX-PEG-PGlu-cRGD5, (d) 700DX-PEG-PGlu-cRGD15, and (e) 700DX-PEG-PGlu-cRAD15 in water (excitation wavelength: 550 nm).

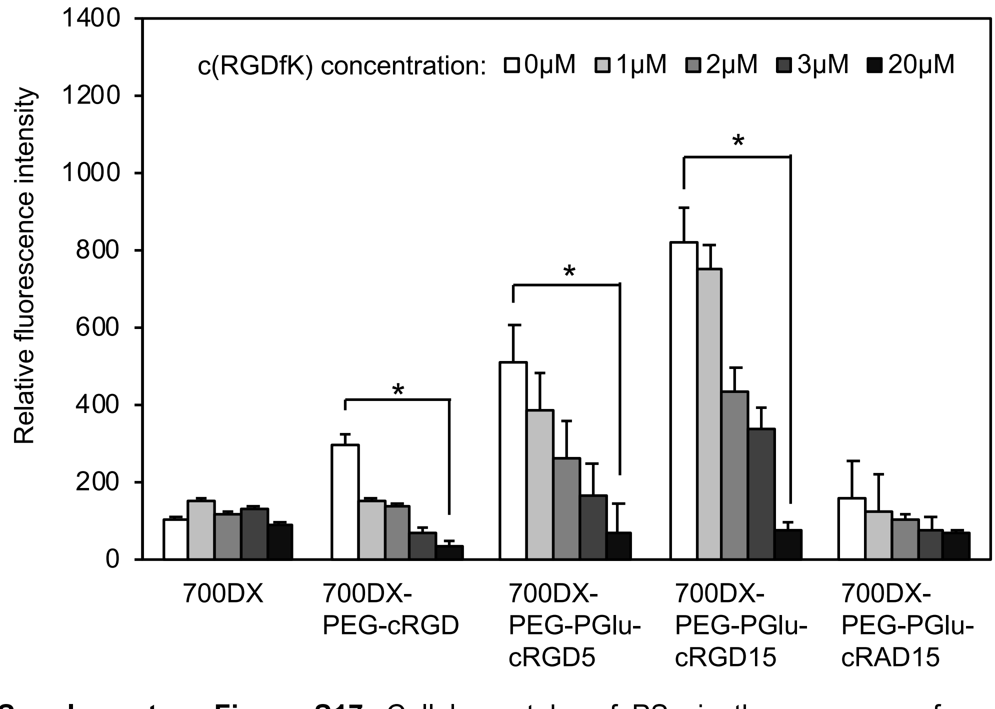

Supplementary Figure S17. Cellular uptake of PSs in the presence of excess free cRGDfK. U87MG cells were incubated with the PSs (200 nM of 700DX) and free cRGDfK (0–20 μM) for 7 h. The results are expressed as mean±S.D. (n=6). Statistical significance was evaluated using one-way ANOVA (*p<0.01).

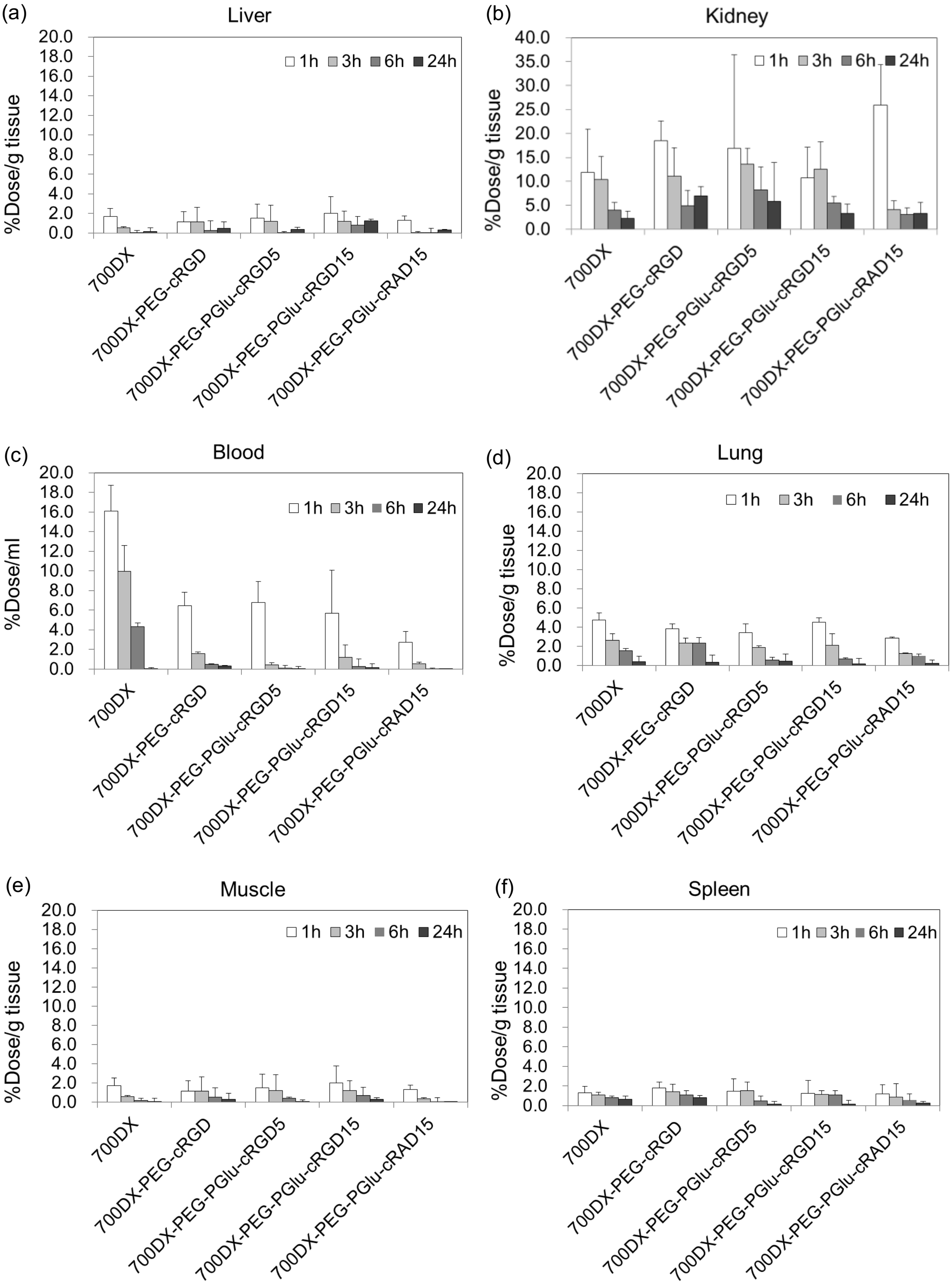
(a)
Liver
(b)
Kidney
(c)
Blood
(d)
Lung
(e)
Muscle
(f)
Spleen
1h 3h 6h 24h
%Dose/g tissue
%Dose/ml
700DX
700DX-PEG-cRGD
700DX-PEG-PGlu-cRGD5
700DX-PEG-PGlu-cRGD15
700DX-PEG-PGlu-cRAD15

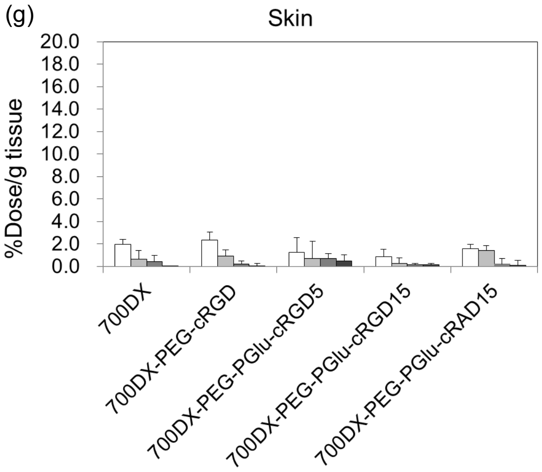

Supplementary Figure S18. Biodistribution study. Accumulation of PSs in (a) liver, (b) kidney, (c) blood, (d) lung, (e) muscle, (f) spleen, and (g) skin was quantified by fluorescence intensity and expressed as a percentage of the injected %dose/g tissue and %dose/mL blood. PSs were injected into mice bearing U87MG tumors (10 μg 700DX/mouse). Tissues were taken out from the mice and gently washed by PBS at indicated time points and homogenized. Fluorescence intensity of homogenized tissue solution was measured (Ex=710nm, Em=780nm). The results are expressed as mean±S.D. (n=4).

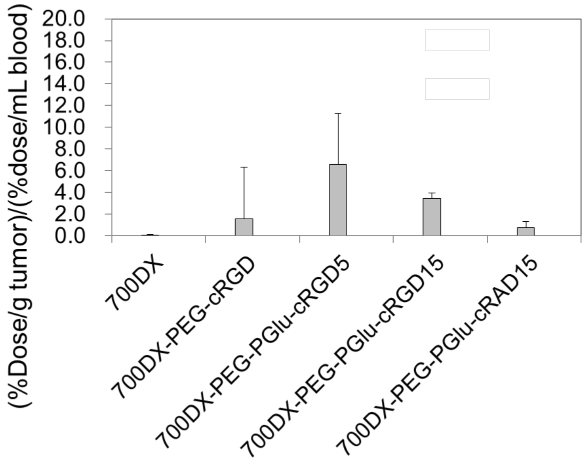

Supplementary Figure S19. Tumor/blood accumulation ratio of PSs 3h after injection. The results are expressed as mean±S.D. (n=4).

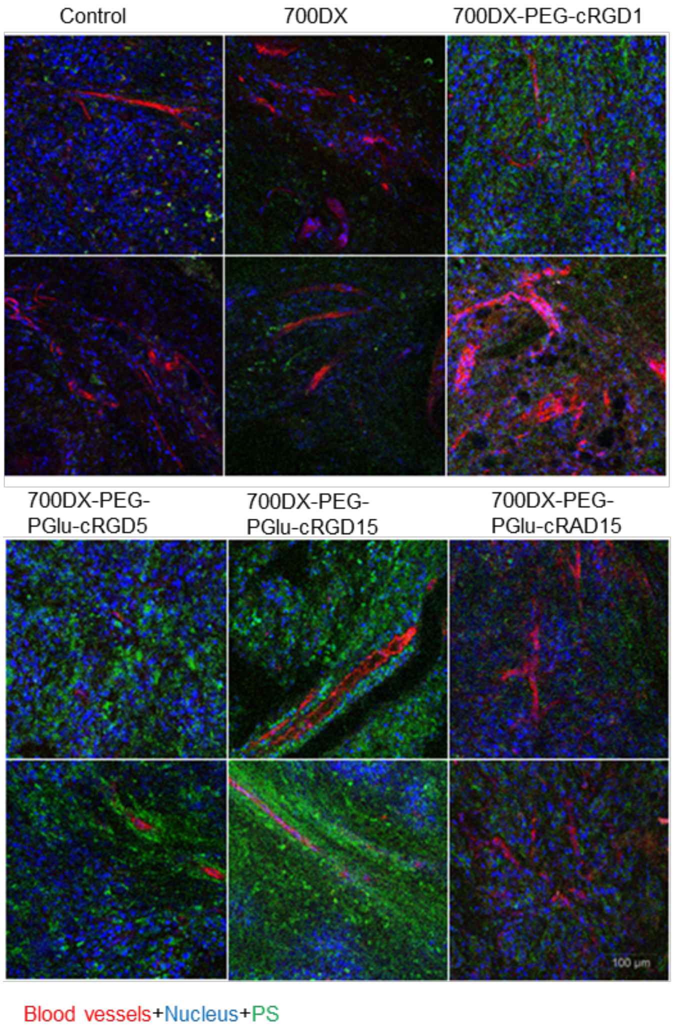

Supplementary Figure S20. Intratumoral distribution of PSs.
